# High-Speed Single-Molecule Tracking of CXCL13 in the B-Follicle

**DOI:** 10.3389/fimmu.2018.01073

**Published:** 2018-05-22

**Authors:** Helen Miller, Jason Cosgrove, Adam J. M. Wollman, Emily Taylor, Zhaokun Zhou, Peter J. O’Toole, Mark C. Coles, Mark C. Leake

**Affiliations:** ^1^Department of Physics, University of York, York, United Kingdom; ^2^Clarendon Laboratory, Department of Physics, University of Oxford, Oxford, United Kingdom; ^3^Centre of Immunology and Infection, University of York, York, United Kingdom; ^4^Department of Biology, University of York, York, United Kingdom; ^5^Department of Electronics, University of York, York, United Kingdom; ^6^Bioscience Technology Facility, University of York, York, United Kingdom; ^7^Kennedy Institute of Rheumatology, University of Oxford, Oxford, United Kingdom

**Keywords:** single-molecule imaging, single-molecule tracking, chemokines, biophysics, lymphoid tissues

## Abstract

Soluble factors are an essential means of communication between cells and their environment. However, many molecules readily interact with extracellular matrix components, giving rise to multiple modes of diffusion. The molecular quantification of diffusion *in situ* is thus a challenging imaging frontier, requiring very high spatial and temporal resolution. Overcoming this methodological barrier is key to understanding the precise spatial patterning of the extracellular factors that regulate immune function. To address this, we have developed a high-speed light microscopy system capable of millisecond sampling in *ex vivo* tissue samples and submillisecond sampling in controlled *in vitro* samples to characterize molecular diffusion in a range of complex microenvironments. We demonstrate that this method outperforms competing tools for determining molecular mobility of fluorescence correlation spectroscopy (FCS) and fluorescence recovery after photobleaching (FRAP) for evaluation of diffusion. We then apply this approach to study the chemokine CXCL13, a key determinant of lymphoid tissue architecture, and B-cell-mediated immunity. Super-resolution single-molecule tracking of fluorescently labeled CCL19 and CXCL13 in collagen matrix was used to assess the heterogeneity of chemokine mobility behaviors, with results indicating an immobile fraction and a mobile fraction for both molecules, with distinct diffusion rates of 8.4 ± 0.2 and 6.2 ± 0.3 µm^2^s^−1^, respectively. To better understand mobility behaviors *in situ*, we analyzed CXCL13-AF647 diffusion in murine lymph node tissue sections and observed both an immobile fraction and a mobile fraction with an example diffusion coefficient of 6.6 ± 0.4 µm^2^s^−1^, suggesting that mobility within the follicle is also multimodal. In quantitatively studying mobility behaviors at the molecular level, we have obtained an increased understanding of CXCL13 bioavailability within the follicle. Our high-speed single-molecule tracking approach affords a novel perspective from which to understand the mobility of soluble factors relevant to the immune system.

## Introduction

Within the immune system, soluble factors such as chemokines, cytokines, and growth factors drive graded responses to extracellular signals, regulating processes including immune cell recruitment at sites of infection (1), lymphoid tissue formation (2, 3), and the maturation of adaptive immune responses (4). Despite their fundamental importance, the precise spatial distribution of soluble factors within tissues remains unclear, due in part to a dearth of experimental techniques capable of measuring diffusion *in situ*.

The emergence of super-resolution imaging in light microscopy has yielded significant insights into the structure and dynamics of the immune synapse (5), with the potential to elucidate the precise spatial localization of soluble factors within the context of a complex tissue. These methods enable spatial localization of single fluorescent probes more than an order of magnitude better than the standard optical resolution limit of ~250 nm, facilitating direct visualization of dynamic molecular processes. Barriers to using super-resolution for quantifying rapid molecular diffusion in biological processes in the aqueous inter- and intra-cellular regions in tissues include poor temporal resolution, due to constraints imposed from limited photon emission, and challenges in data interpretation due to heterogeneous mobility behaviors such as complex underlying diffusion modes or the presence of mixed populations of molecules in multimeric forms.

To achieve the most rapid sampling possible, traditional single-molecule fluorescence tracking techniques must compromise on the image quality or on the type of probe used. Elastic and interferometric scattering can overcome poor fluorophore photophysics to enable rapid sampling; however, they either use relatively large probes that exhibit steric hindrance, or achieve poor specificity in heterogeneous sample environments unless used in conjunction with fluorescent labeling (6–10). Scanning fluorescence methods such as stimulated emission depletion microscopy (STED (11) are limited to ~1 Hz typical frame rates with faster imaging up to ~1,000 Hz possible by trading image quality (12); MINFLUX imaging (13) can operate at 8,000 localizations per second in bacterial cells, but only tracks one molecule at a time, while widefield approaches such as fast variants of photoactivatable localization microscopy (PALM) (14) and stochastic optical reconstruction microscopy (STORM) (15) have integration times of on the order of tens of milliseconds for individual image frames with full reconstructions commonly taking several seconds. Structured illumination approaches (16, 17) at best have frame rates of several hundred Hz and high-intensity illumination methods have enabled super-resolution imaging in living cells at around millisecond timescales (18, 19). Submillisecond fluorescence imaging has been reported previously using relatively large fluorescent bead probes (20), tracking a single molecule at a time (21), in plasma membranes using fluorescently labeled cholesterol analogs or Fab fragments (22, 23) and at short distances from the coverslip using TIRF and HILO imaging (24). However, these methods encounter significant challenges in data interpretation when samples and mobility are heterogeneous, as encountered in tissues. Our method is the first, to the best of our knowledge, to enable submillisecond molecular tracking using a minimally perturbative nanoscale organic dye reporter in a heterogeneous 3D aqueous environment typical of interstitial regions between cells in tissues.

Single-molecule tracking can be used to measure diffusion coefficients of proteins and molecules in localized regions and offers the opportunity to investigate the heterogeneity one molecule at a time compared with the ensemble technique of fluorescence recovery after photobleaching (FRAP) (25–27) and quasi single-molecule approach of fluorescence correlation spectroscopy (FCS) (28, 29). These three techniques have been compared using proteins present in the plasma membrane of cells (30–33), supported lipid bilayers (33, 34), and giant unilamellar vesicles (34), which are all approximated as 2D surfaces.

**Figure 1:**
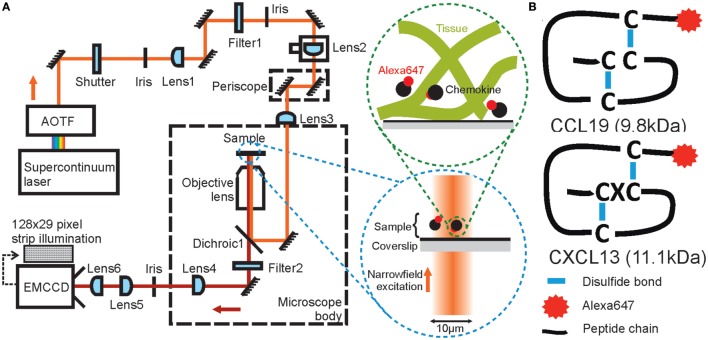
Schematic diagrams of high-speed narrowfield microscopy and the experimental system. **(A)** The imaging framework showing the bespoke fluorescence microscope and diagrams of image acquisition. **(B)** The structure of Alexa Fluor 647 labeled CCL19 and CXCL13.

In this study, we use single-molecule imaging approaches to quantify the diffusion of the chemotactic cytokines (chemokines) CXCL13 and CCL19 (Figure 1B). These molecules are key regulators of lymphocyte migration that are present in spatially distinct regions of the lymphoid tissues such as the lymph node (4). Chemokines are small proteins (~10kDa) that bind G-protein Coupled Receptors (GPCRs) leading to polarization of the actomyosin cytoskeleton and directed migration along localized concentration gradients (35). Chemokine bioavailability is regulated across broad spatiotemporal scales, making direct visualization of these molecules *in situ* challenging. Chemokines are secreted within a dense, heterogeneous microenvironment and undergo transient interactions with their cognate GPCRs and components of the extracellular matrix (ECM) before undergoing receptor-mediated scavenging or enzymatic degradation (35–37). In addition, chemokines are heterogeneous in their binding affinities and are subject to multimerization effects; characteristics that may alter their mobility (38, 39). Simplified hydrodynamic predications (40) employing estimations for the Stokes radius of chemokines and the fluid environment viscosity suggest that chemokine diffusion in hypothetically homogeneous intracellular media in the absence of binding effects would be rapid at ~150 μm^2^ s^−1^, implying ~50 s for a single molecule to diffuse across a 200-µm diameter region of lymphoid tissue. However, this estimate is likely to be a poor predictor of diffusivity as it does not account for dynamic molecular interactions encountered in dense, heterogeneous tissues.

In the following sections, we describe a method to overcome previous technological barriers to the study of molecular mobility *in situ*. Specifically, we have adapted a standard inverted epifluorescence microscope, making important modifications to facilitate minimally perturbative submillisecond single-molecule tracking of rapidly diffusing fluorescently labeled biomolecules *via* subdiffraction limit localizations, and developed bespoke software for precise quantification of underlying molecular mobility of tracked particles. We compared FCS, FRAP, and single-molecule tracking on the well-characterized test system for molecular mobility of bovine serum albumin (BSA), labeled with Alexa Fluor 647 (AF647). We then applied our method to quantify the diffusion of CCL19 and CXCL13 (Figure 1B), in a range of environments of increasing complexity comprising (i) buffer alone and in the presence of the highly branched polysaccharide Ficoll to vary the fluid environment viscosity, (ii) the presence of either surface-immobilized heparan sulfate, or a collagen gel matrix, and further (iii) AF647-tagged CXCL13 was tracked in an *ex vivo* native mouse lymph node environment. Our data suggest that CCL19 and CXCL13 have distinct diffusion rates, and that CXCL13 exhibits both specific binding and diffusion at 6.6 ± 0.4 µm^2^s^−1^ within example sections of the B-cell follicle.

## Results

### Overview of the High-Speed Single-Molecule Tracking Methodology

To enable precise localization and tracking of rapidly diffusing biomolecules, we modified the optical path of a standard inverted epifluorescence microscope (Figure 1A) to implement a broadband laser whose output was selectable over wavelengths ~400–2,000 nm (Figure S1 in Supplementary Material), spanning the excitation spectra of visible-light and near-infrared fluorophores; the beam was de-expanded using a series of lenses to generate a narrow illumination field of ~12 μm full-width at half maximum (FWHM) which could be switched from epifluorescence into total internal reflection fluorescence (TIRF) by controllable translation of a lens, although TIRF was not used in this work. High-contrast epifluorescence images magnified to 120 nm/pixel were captured by an ultrasensitive back-illuminated EMCCD detector (860 iXon + , Andor Technology Ltd.) which could be subarrayed to 29 × 128 pixels to enable rapid frame rates of 1,515 Hz. Images were analyzed using bespoke software (41) written in MATLAB (Mathworks), which enabled automated 2D submillisecond tracking of single fluorescent dye molecules and determination of the microscopic diffusion coefficient *D* from the measured mean square displacement (42). Diffusion coefficients were found by an interative fitting procedure developed with simulated data.

### Speed of Tracking and Image Analysis

A range of sample exposure times of 0.44–1.98 ms per frame were used, with most data acquired at 0.59 ms per frame (0.65 ms cycle time) as a compromise between sampling speed and localization precision. In all cases, we were able to resolve distinct diffusing fluorescent foci of measured 2D half-width at half maximum in the range of 250–300 nm, consistent with single point spread function (PSF) images. Automated foci tracking was utilized for the determination of molecular diffusivity. Foci could be tracked continuously in 2D with ~40 nm precision (Figure S4 in Supplementary Material).

The presence of single molecules was determined by the observation of stepwise photobleaching steps. Examples of this are shown in Figures S1C,D in Supplementary Material. From the manufacturer’s specifications, BSA-AF647 was expected to be labeled with between 3 and 6 AF647 dye molecules and the chemokines were expected to be singly labeled. Only single-molecule bleaching steps were observed in the chemokine data.

The initial intensity of observed foci of the AF647 dye molecule was measured in five conditions: CCL19-AF647 and CXCL13-AF647 in collagen and under heparan sulfate immobilization, and BSA-AF647 in 10% Ficoll. The kernel density estimate was found from the intercept of a line fitted to the first three intensity values measured. Within experimental error the initial intensity for all conditions fell in the 2,000–3,000 count range. The initial intensity is expected to vary for each condition due to the different local environment of the AF647 tag on each molecule, including different allowed orientations and varying flexibility of the linker. Furthermore, the viscosity of the medium is known to affect the emission profile of AF647 and will cause slight variations in total measured intensity due to the use of a band pass filter in the emission path.

**Figure 2:**
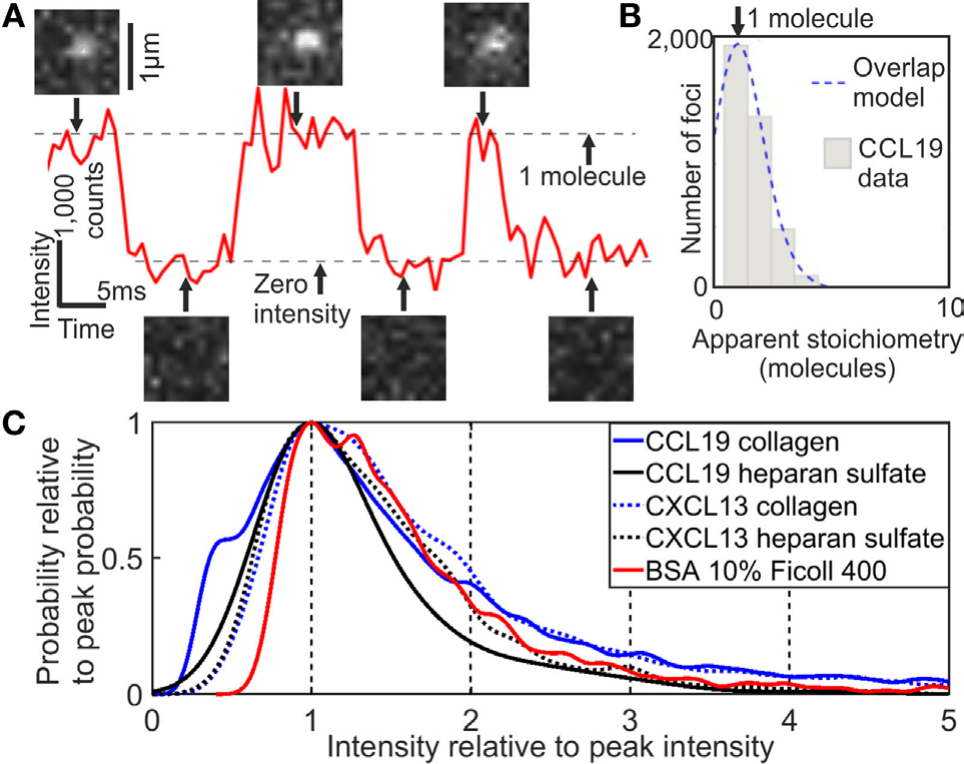
Single-molecule stoichiometry of CCL19-AF647. **(A)** Tracking of photoblinking Alexa Fluor 647 (AF647): localizations and intensity over time with sample images from the acquisition. **(B)** Distribution of apparent CCL19 foci stoichiometry (gray) overlaid with the predicted distribution based on randomly overlapping point spread functions (blue). **(C)** Kernel density estimates of intensity of AF647 labeled CCL19 in collagen (solid blue line), and under heparan sulfate immobilization (solid black line); CXCL13 in collagen (dotted blue line), and under heparan sulfate immobilization (dotted black line); and bovine serum albumin in 10% Ficoll (solid red line). All traces are normalized to the primary peak for clarity (see Supplementary Material).

Analysis of the tracking data from CXCL13-AF647 and CCL19-AF647 using step-wise dye photobleaching showed predominantly monomeric populations for each (Figure 2; Figure S1 in Supplementary Material). Apparent stoichiometry values determined from the intensity of tracked fluorescent foci greater than one molecule per foci may be due to real multimeric complexes or potentially due to the overlap of monomeric foci images in the 2D projection that is imaged, especially in the case of high dye concentration. The maximum number of detected foci in one frame of 15 foci in our case was used in a random overlap model which assumes a Poisson distribution for nearest neighbor foci overlap probability (43, 44). This analysis indicates an 18% probability for random single foci overlap. The predicted intensity of foci due to random overlap could be obtained by convolving the intensity distribution of a single molecule of AF647 (width scaled by the square root of the apparent stoichiometry) with the apparent stoichiometry distribution generated by the random overlap probability prediction. The overlap model was found to be statistically identical to the experimentally observed distribution below apparent stoichiometry values of six AF647 molecules per foci (*p* < 0.05, Pearson’s χ^2^ test). A small proportion of less than 5% of foci we found to have a higher apparent stoichiometry than that predicted from the random overlap model; it is possible that these may be indicative of some additional factors not captured in the basic overlap model such as non-uniformity in illumination across the field of view and experimental autofluorescence.

### Iterative Data Simulation and Experimental Data Fitting

Our analysis of the distribution of effective diffusion coefficients obtained from the single-molecule tracking data was corroborated through simulations of diffusing and immobilized foci using realistic signal and background noise values. Iterative cycles of simulation and experimental data fitting were used to determine initial parameters for fitting and the form of the fitting functions. All simulations were run and fitted with and without the addition of Gaussian white noise. The first simulated values were chosen by fitting the experimental data with a two gamma distribution model (45) to account for two diffusive populations.

**Figure 3:**
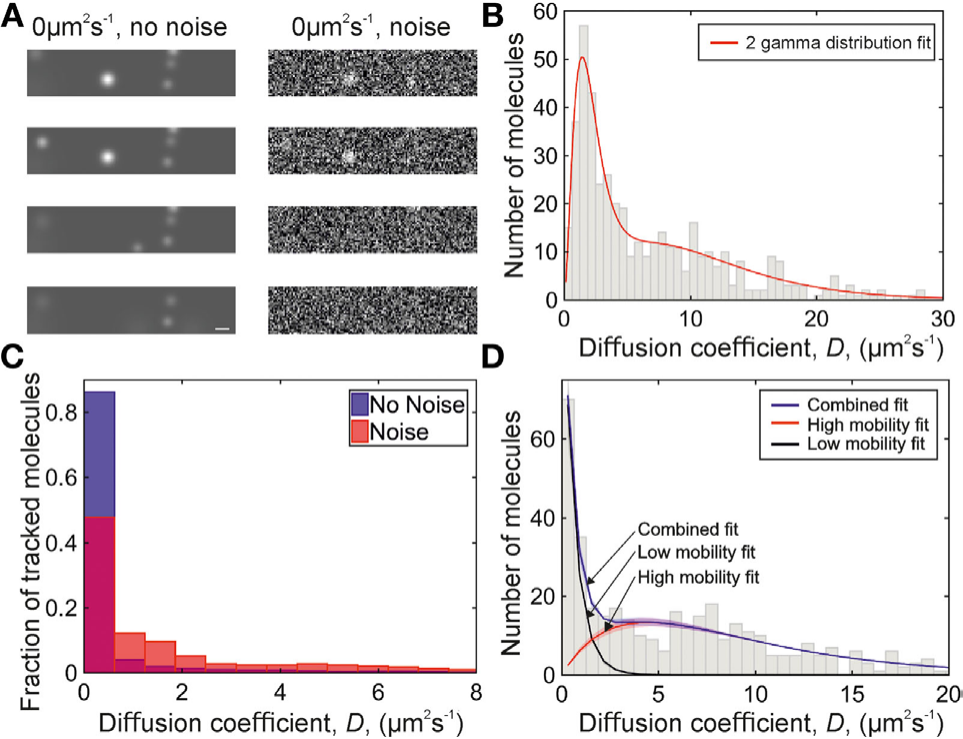
Simulations of chemokine data. **(A)** Sample simulation images, shown with and without Gaussian white noise added. Scale bar 1 µm. **(B)** Two-gamma distribution fit (red) to diffusion coefficients found from a simulation of 1.6 and 10 µm^2^s^−1^ data with Gaussian white noise. **(C)** Histograms showing the distribution of simulated 0 µm^2^s^−1^ data with (red, overlaid) and without (blue) Gaussian white noise. **(D)** Diffusion coefficient distribution from a simulation of 0 and 9 µm^2^s^−1^ data with Gaussian white noise. Fitted populations are shown in black for the immobile, red for the mobile, and blue for the combined fit. Shaded areas indicate one SD.

Initially two distributions, 1.6 µm^2^s^−1^ and a 50:50 mixed population of 1.6 and 10 µm^2^s^−1^, were simulated (sample images in Figure 3A). The data were plotted *via* kernel density estimation, and fitted with 1, 2, and 3 gamma distribution functions with four independent steps, all parameters constrained to be positive, each term was multiplied by a fractional prefactor to preserve the unity area of the kernel density plot, and the χ^2^ goodness-of-fit parameter was evaluated (Table S1 in Supplementary Material). The χ^2^ statistic accounts for the number of free parameters in the fit, and is used to determine if decreasing residuals are caused by increasing the number of free parameters. For the one component distribution, the one-gamma fit gave the lowest χ^2^, and for the two-component distribution, the two gamma fit gave the lowest χ^2^ value, as expected. Applying these three models to the experimental collagen and heparan sulfate immobilized chemokine data gave the lowest χ^2^ for a two-component fit, except for CCL19 in heparan sulfate, which contained a very low proportion of mobile tracks and was well fitted by a single-component distribution. From this, it was determined that a two-component distribution should be fitted to the experimental data.

The number of independent steps is usually taken to be the same as the number of steps analyzed in gamma distribution fitting analysis of microscopic diffusion coefficients, where it is a parameter governing the shape of the distribution. Strictly, steps are only independent when non-overlapping steps are used (45), but when the localization precision (in nm) of single particles is small compared with the distance moved between localizations in a track the diffusion coefficient distributions from overlapping steps are still well-approximated by the assumption of independent steps. In this work, the temporal resolution is increased to a level where the localization precision is no longer negligible compared with the distance moved between localizations, and steps containing the same localizations are no longer well approximated as being independent. To investigate the independence of the steps in the data and determine the relevant fitting parameter, simulations of 1.6 and 10 µm^2^s^−1^ were made separately, and fitted with a single-component gamma distribution where the number of independent steps was allowed to vary. The results (Table 1) indicate this value to be around two, in line with expectations of consecutive steps containing the same localization not being independent, reducing the number of steps by half.

**Table 1:** Results of one-gamma distribution fitting to simulated single diffusion coefficient distributions.

Simulated condition	Number of tracks	Fitted value of D (μm^2^s^−1)^	Fitted value of*N*(μm^2^s^−1)^	*R*^2^ value of fit
1.6 µm^2^s^−1^, no noise	1,579	1.72 (1.68, 1.76)	2.24 (2.15, 2.33)	0.9892
1.6 µm^2^s^−1^, noise	401	2.19 (2.12, 2.25)	1.75 (1.67, 1.83)	0.9777
10 µm^2^s^−1^, no noise	1,519	10.21 (9.77, 10.6)	2.27 (2.08, 2.45)	0.9343
10 µm^2^s^−1^, noise	463	10.04 (9.53, 10.54)	2.77 (2.47, 3.08)	0.8968

*Noise or no noise refers to the presence of Gaussian white noise proportional to the intensity in the simulation. 95% confidence intervals are given in brackets*.

**Figure 4:**
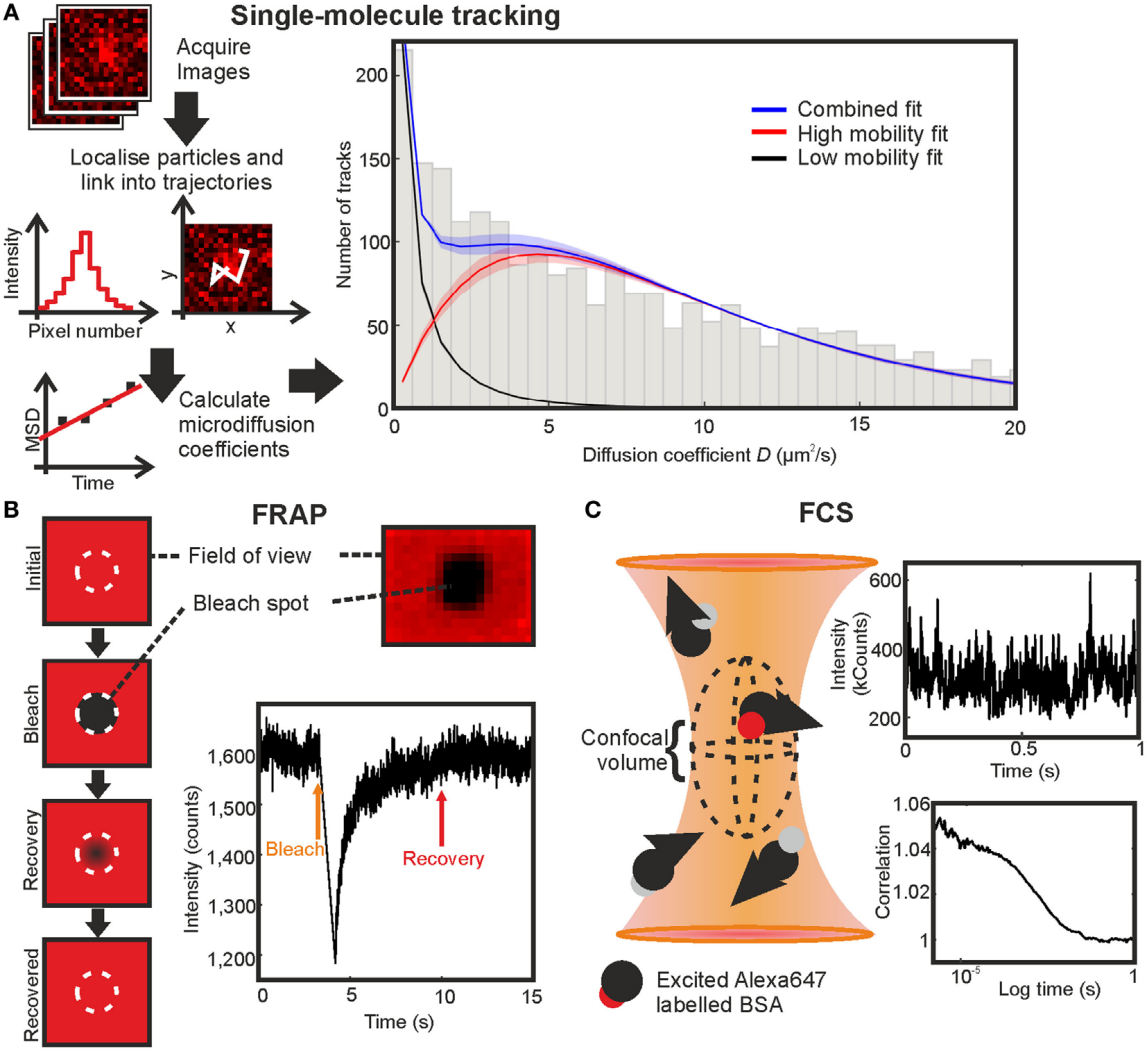
Comparing fluorescence recovery after photobleaching (FRAP), fluorescence correlation spectroscopy (FCS), and single-molecule tracking on BSA-AF647 in 10% Ficoll 400. **(A)** Single-molecule tracking: simplified schematic of the stages in tracking and the resulting fit with shaded regions indicating error bounds of one SD. **(B)** FRAP: schematic of technique, profile of bleached region in an immobilized sample, and example fluorescence intensity recovery trace. **(C)** FCS: schematic of the confocal volume, example section of intensity fluctuation trace, and correlation curve.

**Figure 5:**
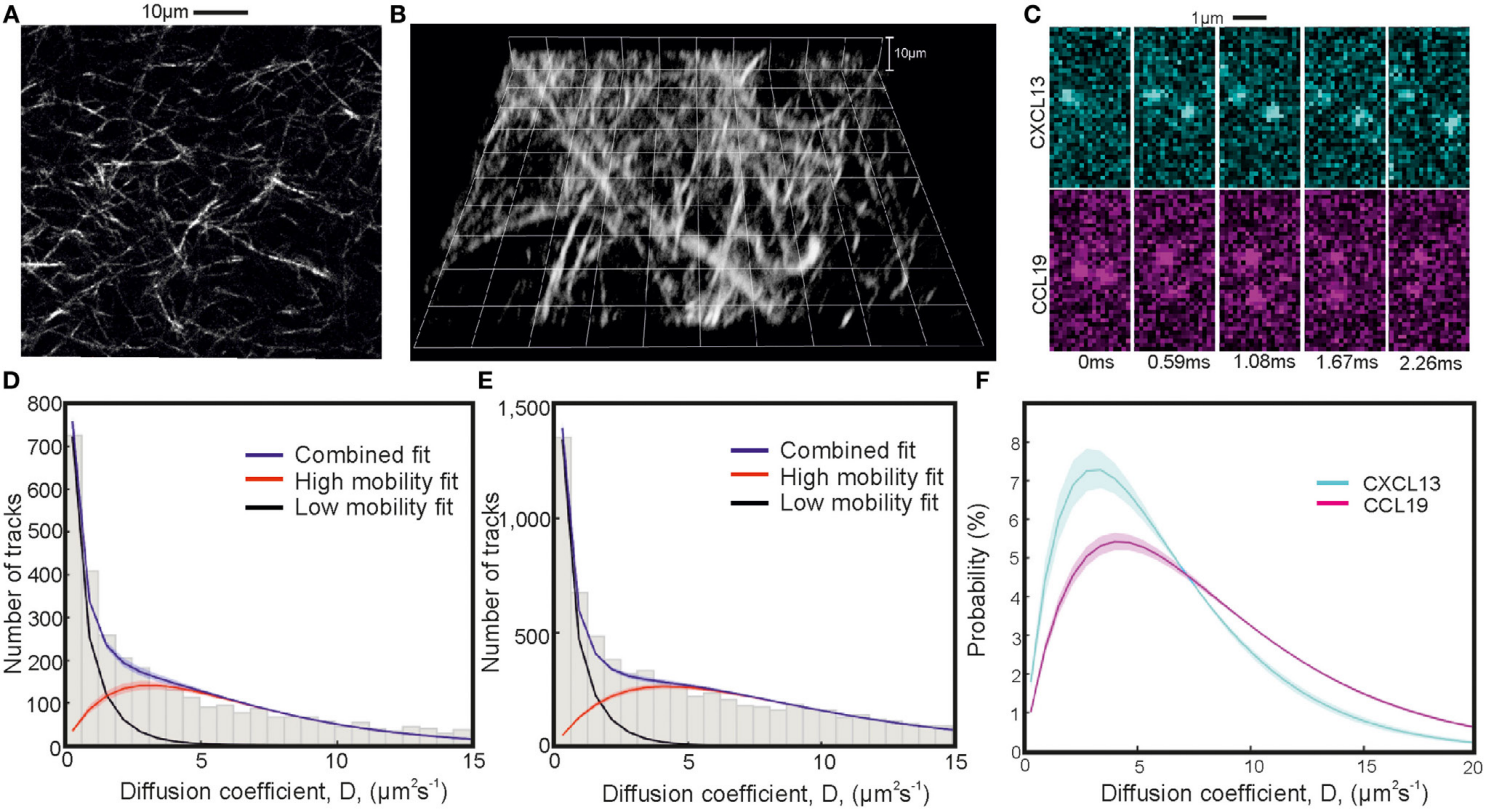
Single-particle tracking of chemokines in collagen. Second-harmonic imaging microscopy (SHIM) of collagen network in **(A)** 2D and **(B)** 3D. **(C)** Representative consecutive submillisecond images of chemokines in collagen. **(D,E)** Fitted diffusion coefficient distribution of CXCL13-AF647 and CCL19-AF647 showing mobile and immobile components in a collagen matrix with **(F)** just the fitted high-mobility diffusion coefficient distributions of CXCL13-AF647 (cyan) and CCL19-AF647 (magenta) (shaded areas indicate one SD).

Fitting simulations of a 50:50 population of molecules with diffusion coefficients of 1.6 and 10 µm^2^s^−1^ with two gamma distributions with the same fitted value of *N* in each distribution, and *N* constrained to be in the range 0–4 (Figure 3B; Table S2 in Supplementary Material), gives a distribution which does not match the experimental data (Figure 4A and 5D,E): when the data are placed in a histogram based on measured diffusion coefficient the experimental data show a peak in the first bin width, which is not seen in the simulation of 1.6 µm^2^s^−1^ data. The 1.6 µm^2^s^−1^ data were simulated because these were found as a preliminary result of fitting to the experimental data, but a simulation of truly immobile data with a diffusion coefficient of 0 µm^2^s^−1^ gave a peak in the first bin of the histogram when put into bins with the width of the localization precision (see Figure 3C), matching the experimental data.

Our simulated particle diffusion analysis suggests that the low-mobility population in the experimental data is immobile at the level of the localization precision. Fitting the distribution of simulated 0 µm^2^s^−1^ data with a single-gamma distribution gave a value of *N* less than 1, and requires the fit applied to the experimental data to include a different value of *N* in the distribution fitted to each population with the value of *N* being less than 1 for the low-mobility population, and 2 for the higher mobility population.

Applying this fit, with the constraint that the diffusion coefficient of the immobile population must fall within the first bin of the histogram, gave the fitted experimental diffusion coefficients. To qualitatively compare the simulated and experimental data, a mixed simulation of 0 and 9 µm^2^s^−1^ data with Gaussian white noise was performed and fitted in the same way, giving a diffusion coefficient for the mobile peak of 8.9 ± 0.4 µm^2^s^−1^. The distribution is similar in profile to the data for CCL19-AF647 in collagen (see Figure 3D).

### Diffusion in Buffer and Ficoll Solutions

In PBS buffer alone, the chemokine diffusion was in general too fast to track over consecutive image frames (Video S1 in Supplementary Material). While this is consistent with theoretical estimates using the Stokes–Einstein relation which gives *D* ~150 μm^2^ s^−1^ for chemokines in an aqueous environment, the application of 10% Ficoll increased the fluid viscosity by a factor of 5.6 to 0.005 Pa s^−1^, which enabled single-particle tracking; if diffusion had been well modeled by the Stokes–Einstein relation the diffusion coefficients in the higher viscosity Ficoll solution would be expected to be ~30 μm^2^ s^−1^ and particles would still not be tracked. The ability to track single chemokines in a medium of this viscosity demonstrates that the theory is not adequate to describe chemokine diffusion and motivates our experimental measurements.

The experimental system was tested first on a non-chemokine control of AF647-tagged BSA (BSA-AF647). The results of single-particle tracking of BSA-AF647 were consistent with a proportion of immobile tracks associated with the coverslip surface and a freely diffusing mobile population with *D*_mobile_ = 9.3 ± 0.4 µm^2^s^−1^ (Figure 4; Videos S2 and S3 in Supplementary Material). Including theoretical expectations based on hydrodynamic modeling of BSA as a Stokes sphere of radius 3.48 nm for monomeric BSA, with a monomer to dimer ratio of 15:2 (measured using SEC-MALLS quantification, Figure S2 and Table S3 in Supplementary Material) and incorporating Faxen’s law for distances of 10 nm, at which distances increased viscosity effects occur at the coverslip boundary (46), the fitted mobile value is found to be in agreement with the theoretical value of 9.4 µm^2^s^−1^.

### Comparison of SMT With FCS and FRAP

The diffusion coefficient of AF647 labeled BSA in a Ficoll solution was additionally measured with FCS and FRAP to generate a comparison of the three methods in a complex, non-surface environment (Figures 3A–E in Supplementary Material). FRAP and FCS gave diffusion coefficients of 7.1 ± 0.3 and 18.8 ± 0.3 µm^2^s^−1^, respectively (Figures 4B,C; Table 2). The values found for the diffusion coefficients by these methods are summarized in Table 1 with the number of traces used for each measurement. The result for FCS is higher than the theoretical value, while that for FRAP is significantly lower even considering Faxen’s law, temperature fluctuation, and non-monomer content in BSA measured by SEC-MALLS (Figure S2 and Table S3 in Supplementary Material); however, the effects of using an axially thin sample and including only 2D recovery in the FRAP analysis were not accounted for.

**Table 2:** Measurements of the diffusion coefficient of Alexa-647 labeled BSA in 10% Ficoll 400.

Condition	Diffusion coefficient (μm^2^s^−1^)	Number of measurements
Theoretical with stokes radius 3.48 nm	12.3 ± 0.1	
FCS	18.8 ± 0.3	27 traces
FRAP	7.1 ± 0.3	30 repeats
Single-molecule tracking	9.3 ± 0.4	2,608 tracks (fitted 1,113 mobile tracks)

*Variation on the theoretical value is due to a potential ±2°C temperature change in the laboratory*.

The FRAP and FCS results differ by a factor of 2.6, in general agreement with previous results from others in which diffusion coefficients found by FCS are mostly higher than those found by FRAP (34), with FCS giving values up to an order of magnitude higher than the values found by FRAP (30, 31) and often attributed to the different spatial scales of the two measurements or the high number of assumptions required in fitting FRAP data (33), such as the profile of the bleaching laser, which are likely to be factors in the experiments performed here. The value found by SMT was the closest to the theoretical estimate of the diffusion coefficient in Ficoll of the experimental viscosity.

Fluorescence correlation spectroscopy and FRAP were also performed on the chemokines in 10% Ficoll 400. FCS produced autocorrelation curves with similar amplitude to BSA-AF647, but high variation was observed in the relative sizes and characteristic decay times of the triplet and translational diffusion populations (Figure S3F in Supplementary Material), resulting in no consensus value of the diffusion coefficient. Both FCS and FRAP measurements were hindered by the presence of large multimers of chemokine (Figure 3G in Supplementary Material). Multimers of this type are simply avoided by visual identification in single-molecule tracking experiments.

### Diffusion Coefficients of CXCL13 and CCL19 in Collagen

The values of the diffusion coefficients were determined in collagen reconstituted from rat tails to produce a simplified tissue mimic. The structure of the collagen was checked for the required formation of non-centrosymmetric structure with second-harmonic imaging microscopy (SHIM) (see Figures 5A,B). The fibril diameters observed are in agreement with those seen by Chen et al. (47), and show qualitatively similar structure.

The values of the diffusion coefficients of CXCL13 and CCL19 in collagen were found by fitting a two gamma distribution to a histogram of the single-molecule tracking data with bin width corresponding to 40nm given by peaks in the localization precision at low diffusion coefficient found from heparan sulfate immobilization of the labeled chemokines (Figures S4A,B; Videos S7 and S8 in Supplementary Material). Heparan sulfate immobilization was verified by an extremely high proportion of immobile tracks (Figures S4C,D in Supplementary Material). The results of the fitting are given in Table 3, and the distributions for each chemokine showing the mobile and immobile populations are shown in Figures 5C–E with sample images shown in Figure 5C. The relative size of the mobile and immobile populations cannot be accurately accounted for as immobile populations were photobleached to enable imaging of highly diffuse mobile populations.

**Table 3:** Diffusion coefficients of CXCL13 and CCL19 in collagen.

AF647 labeled chemokine	Theoretical diffusion coefficients in water (μm^2^s^−1)^	Fitted diffusion coefficient (μm^2^s^−1)^	Error (μm^2^s^−1)^	Number of highly mobile tracks	*R^2^*of combined fit
CXCL13	149	6.2	0.3	1,930	0.980
CCL19	146	8.4	0.2	4,859	0.984

*Optimized values were found by fitting a two gamma distribution to single-molecule tracking data*.

Modeling the submillisecond tracking data as a mixture of immobilized and mobile tracks generated excellent agreement to the experimental data (Table 3). Our findings indicated a higher diffusion coefficient for CCL19-AF647 than for CXCL13-AF647 in the controlled environment of collagen (Figure 5F; Videos S4 and S5 in Supplementary Material), which we measured as 8.4 ± 0.2 and 6.2 ± 0.3 µm^2^s^−1^, respectively. This heterogeneity is consistent with molecular mass expectations and may contribute to the formation of distinct spatial patterning profiles *in situ*.

### Binding of CXCL13 to Lymph Node Tissue Sections

The experiments with BSA-AF647, CCL19-AF647, and CXCL13-AF647 in collagen suggested a 4–6 times higher proportion of molecules in the immobile fraction than the mobile fraction for the chemokines than for BSA-AF647, even allowing for differences introduced by utilizing a pre-bleach to increase the fraction of mobile tracks. This is in agreement with previous results: CXCL13 is secreted in soluble form, but is known to interact with ECM components (48) and thus is likely to comprise a significant immobile fraction. To assess both fractions while also ensuring a sufficiently low concentration of CXCL13-AF647 to enable single-molecule detection, we incubated murine lymph node tissue cryosections with CXCL13-AF647 and performed a short wash step. While removing a large component of the soluble fraction of CXCL13-AF647, this preparation facilitated tracking of both mobile and immobile fractions of CXCL13-AF647 *in situ*, depending on the microscopy method employed.

**Figure 6:**
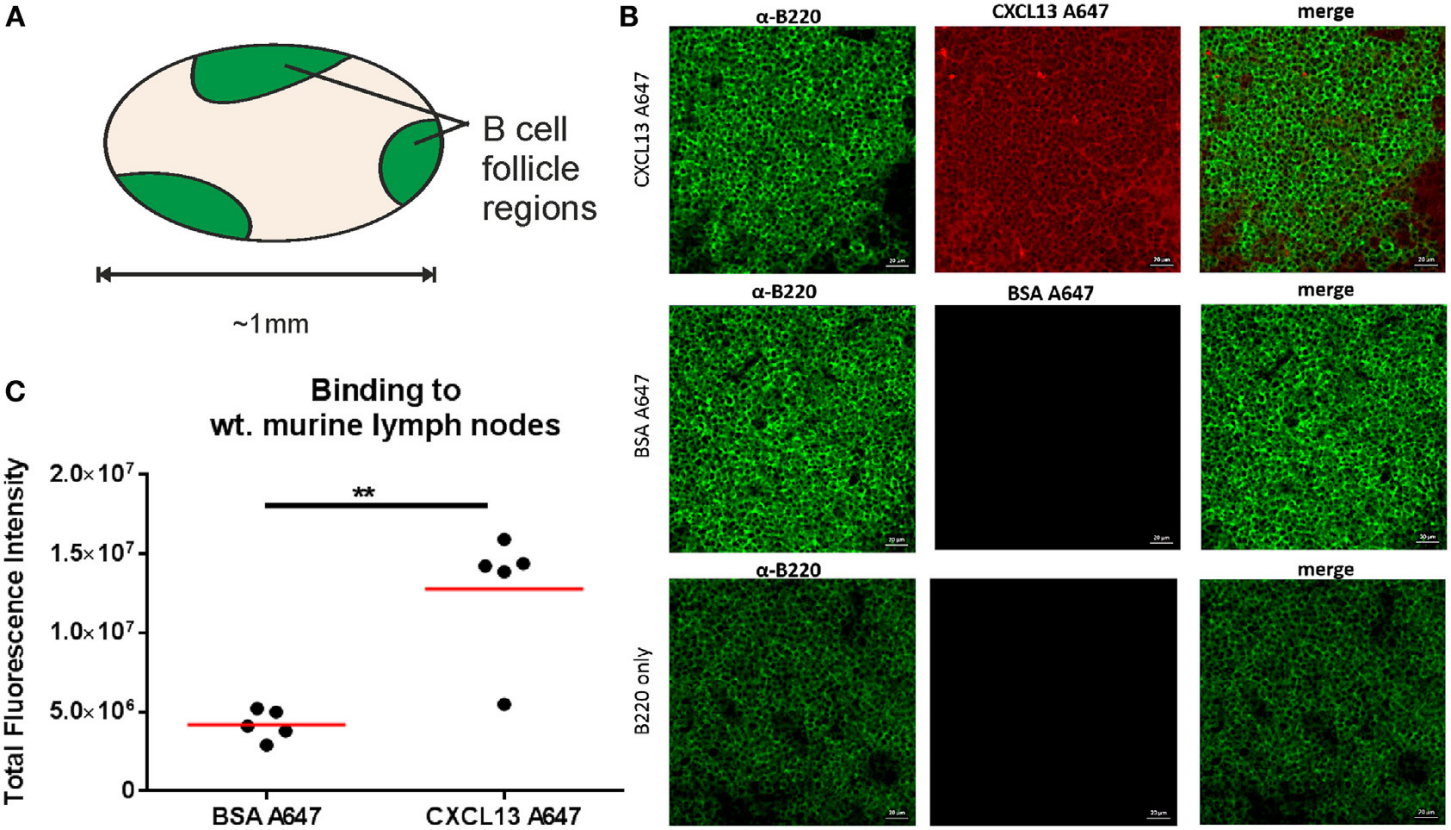
Confocal microscopy quantification of CXCL13-AF647 binding to lymph node follicles. **(A)** Schematic diagram of approximate locations of B-cell follicles in a wild-type murine lymph node. **(B)** Exemplar confocal microscopy images of CXCL13-AF647 and BSA-AF647 binding to lymph node tissue follicles (B220 + regions of lymph node tissue sections), and control with only B220 staining. **(C)** Quantification of the total fluorescent intensity for a fixed size imaging plane within a lymph node follicle. Each data point represents a distinct follicle.

To assess the specificity of binding, we used confocal microscopy to quantify the fluorescent intensity of B220^+^ regions (Figure 6A) of lymph node tissue sections that had been incubated with either CXCL13-AF647 or BSA-AF647. The fluorescent intensity values obtained were significantly higher for samples incubated with CXCL13-AF647 (Figures 6B,C), suggesting that the binding of CXCL13 to lymph node follicles was specific.

**Figure 7:**
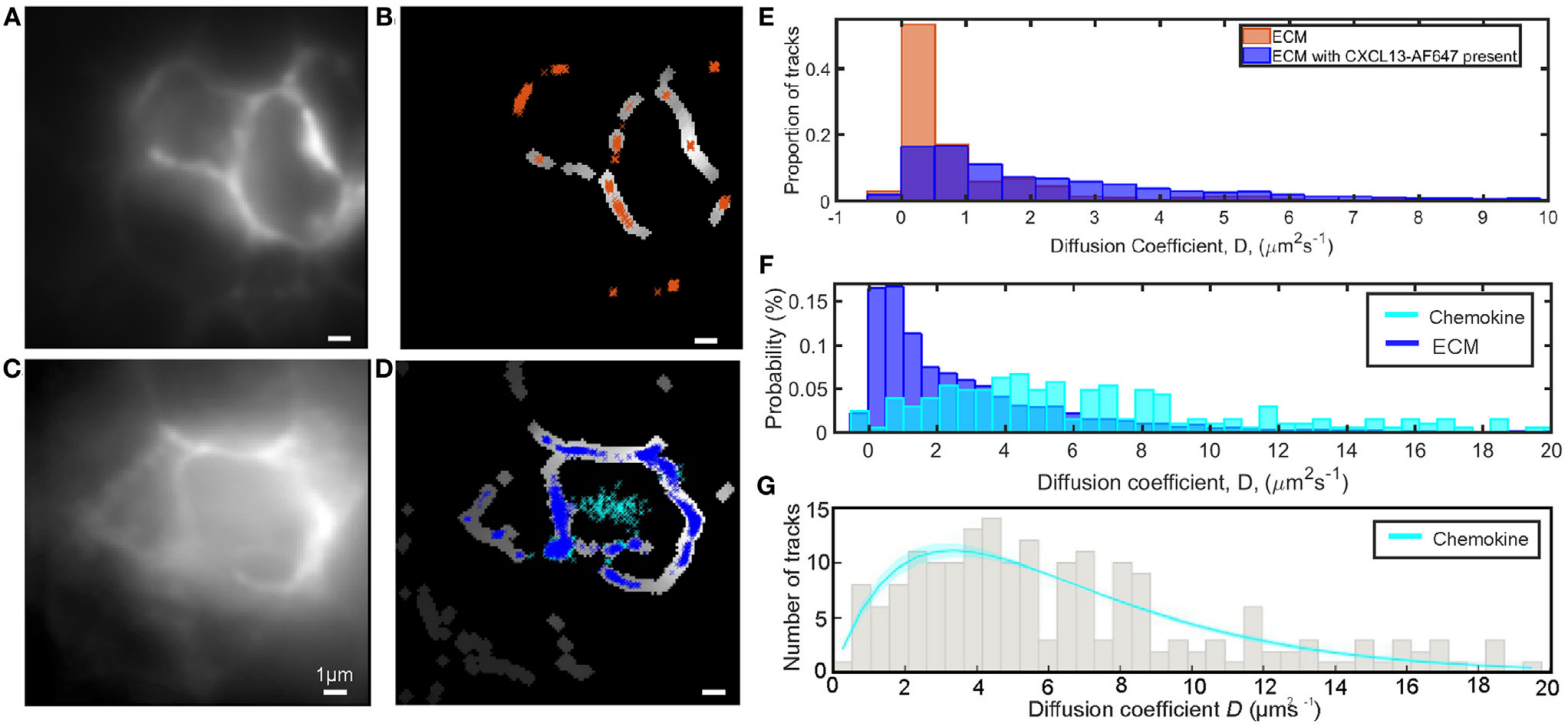
Single-molecule analysis of CXCL13-AF647 in tissue. **(A)** Intensity average image of image acquisition to show autofluorescent extracellular matrix (ECM) in B220-stained B-cell follicle with no added chemokine. **(B)** Areas of **(A)** identified as ECM by segmentation with overlaid track localizations colored orange. **(C)** Intensity average image of image acquisition to show autofluorescent ECM in B220-stained B-cell follicle with added chemokine. **(D)** Areas of **(C)** identified as ECM by segmentation with overlaid track localizations colored by location on ECM (blue) or in the interstitial spaces between cells (cyan). **(E)** Comparison of diffusion coefficients of localizations in ECM locations in the presence (blue) and absence (orange) of CXCL13-AF647 **(F)** Comparison of diffusion coefficients for the ECM (blue) and chemokine (cyan) populations when tracking CXCL13-AF647 in lymph node tissue shown. **(G)** Distribution and fit of chemokine diffusion coefficients of CXC13-AF647 in tissue sections, shaded area indicates one SD.

In the single-molecule microscopy experiments, we imaged and tracked CXCL13-AF647 in B-cell follicles of *ex vivo* murine lymph node tissue sections with super-resolution precision at ~2 ms timescales (Figure 7C; Video S6 in Supplementary Material), determining the precise location in the tissue using FITCB220 (B-cell-specific marker) (Figure S5 in Supplementary Material). Auto-fluorescent ECM components were localized by the tracking software and were segmented to allow discrimination of tracks from the immobile ECM and the diffusing chemokine (Figures 7C,D,F). When the same segmentation analysis was performed on control tissue sections prepared by the same protocol except without the addition of CXCL13-AF647, similar autofluorescent structures were seen and could be segmented (see Figures 7A,B). The observed diffusion coefficient distributions of tracked ECM regions within the B-cell follicle in the presence of chemokine were observed to be skewed toward higher mobility than those in the absence of chemokine (Figure 7E), further confirming the presence of specific binding of CXCL13-AF647 in the ECM regions.

### Diffusion Coefficient of CXCL13 in Lymph Node Tissue Sections

For the same single-molecule imaging experiments performed at ~2 ms timescales described above, after discrimination of mobile and immobile tracks by segmentation, the diffusion coefficient of mobile tracked particles for the field of view shown was fitted with a single-gamma distribution indicating *D* = 6.6 ± 0.4 µm^2^s^−1^ (Figure 7G). Due to the inclusion of a wash step in our sample preparation, we expect the majority of the mobile particles to be in the interstitial spaces between cells, although a small fraction may be within cut cells due to the preparation of tissue sections. The lack of fluorescent localizations in the central gap in the control sample is further evidence that the mobile population observed in the data is CXCL13-AF647.

To validate our result, we performed a simulation of the conditions in the tissue, with the mean background level and SD of the noise taken from the control data. A total of 1,000 frames of data of particles with diffusion coefficient 6.6 µm^2^s^−1^ were generated, with spot intensities and spot widths taken from the experimental data for tracking in tissue. Sample images from the simulation can be seen in Figure S6A in Supplementary Material. The result of fitting this simulation was *D* = 7.0 ± 0.4 µm^2^s^−1^ (Figure S6B in Supplementary Material), in agreement with the simulated value. Whilst interstitial spaces are heterogeneous in their size, crowding, and local viscosity, taken together out results demonstrate the ability of our method to extract diffusion coefficients from challenging experimental data.

## Discussion

In this work, we have demonstrated the application of a high-speed single-molecule tracking microscopy system that is compatible with traditional widefield light microscopes. We compared our method with the traditional methods to measure molecular mobility of FCS and FRAP using one of the standard test molecules for molecular mobility (BSA). We applied this new approach to investigating hitherto unquantified molecular mobility of chemokines in complex environments, finding values of diffusion coefficients of 8.4 ± 0.2 µm^2^s^−1^ for CCL19-AF647 and 6.2 ± 0.3 µm^2^s^−1^ for CXCL13-AF647 in collagen, and of 6.6 ± 0.4 µm^2^s^−1^ for CXCL13-AF647 in example lymph node follicle sections, in addition to identifying a specifically bound CXCL13-AF647 population in the B-cell follicle. While we demonstrate the efficacy of the approach on chemokines, this is a proof-of-concept for a more general scheme that could be applied to signaling lipids and cytokines.

Our method enables single-molecule tracking of organic dye probes at submillisecond timescales, down to less than half a millisecond per imaging frame while still enabling 40-nm localization precision in realistic tissue mimetic microenvironments. In *ex vivo* lymph node tissue sections, we were able to perform rapid super-resolution sampling down to 2 ms per imaging frame and still achieve single-molecule detection sensitivity. To the best of our knowledge, this is the first application of submillisecond tracking of small fluorophores away from the coverslip interface.

We characterized the output of our single-molecule tracking tools with a range of simulations of mixed molecular mobility using realistic levels of signal and noise comparable to those exhibited at challenging single-molecule detection levels with very rapid submillisecond fluorescence sampling. We also tested our imaging and analysis methods using a fluorescently labeled version of the well-characterized molecule, BSA, and compared these with experiments using FRAP and FCS. The values of diffusion coefficient for mobile BSA determined using our rapid SMT method were in close agreement to expectations based on hydrodynamic modeling. Equivalent BSA mobility values estimated from using FRAP or FCS were less reliable. We compared the diffusion coefficients of BSA-AF647 measured by FCS, FRAP, and SMT, producing a comparison of these techniques away from the coverslip interface, and showing agreement in the relation of the measured values with most previous studies performed on lipid bilayers, or on live cell plasma membranes.

We measured the diffusion coefficients of CXCL13-AF647 and CCL19-AF647 in reconstituted collagen, finding values of 6.2 ± 0.3 and 8.4 ± 0.2 µm^2^ s^−1^, respectively. We further measured the diffusion coefficient of CXCL13-AF647 in *ex vivo* lymph node tissue section, finding a value in agreement with that measured in collagen of 6.6 ± 0.4 µm^2^s^−1^. Fluorescent tags increase the mass of the labeled molecule and potentially affect the properties of diffusion. The fluorophore used in this work, AF647, was chosen for its small mass, which is especially important here given the small mass of chemokines. This choice resulted in a ~10% increase in mass of the labeled chemokine compared with the unlabeled chemokine, but this is lower than would have been achieved with other fluorophores. Following the assumptions of the Stokes–Einstein model of a spherical protein of uniform density, a 10% increase in mass would only decrease the observed diffusion coefficient by ~3.2%. We observe a large discrepancy between our empirical measurements for chemokine diffusion rates and the higher estimated values derived on the basis of Stokes–Einstein relation. However, this discrepancy is of the same order of magnitude as that previously observed for the same theoretical calculation for GFP (~93 μm^2^s^−1^) and measured experimental values in *Escherichia coli* [~7.7–9 μm^2^ s^−1^ (49, 50)]. This discrepancy may be indicative of additional factors that affect molecular mobility in tissues but are not captured in the simplistic Stokes–Einstein relation. These factors might include, for example, dynamic physical and chemical processes which result in more constrained mobility such as transient biochemical interactions within the localized microenvironment, as have been observed previously in studies which suggest that CXCL13 binds to ECM components (48).

Placed in an immunological context, our data show that chemokine mobility is multimodal in complex environments. Using our novel imaging approach, we were able to quantitatively identify a mobile and immobile fraction in collagen, and using a combination of confocal microscopy and single-molecule imaging we identified mobile and bound populations of CXCL13-AF647 in lymph node follicles. Thus, it is important to consider the contributions of both populations of CXCL13 upon cellular behaviors, rather than taking a view where it acts in either a soluble or an immobile way. The properties and likely highly constrained nature of CXCL13 diffusion within the follicle may provide an insight into how B cells can form such tightly compartmentalized microanatomical structures such as the follicle, or the germinal center light-zone.

Our novel high-speed microscopy and analysis outperform traditional molecular mobility tools of FRAP and FCS in being able to capture diffusional heterogeneity relevant to real, complex biological systems exemplified by underlying mobile, and immobile states. The high time resolution achieved with our system enables rapid diffusion to be quantified in heterogeneous aqueous environments typical of interstitial regions between cells in tissues, while still retaining super-resolution spatial precision and single-molecule detection sensitivity, enabling new insight into complex systems. A key advantage of our rapid single-particle tracking method is its ability to determine the underlying heterogeneity in the mobility of the molecular population exemplified here by chemokines that diffuse in different environments. While we demonstrate the efficacy of the approach on chemokines, this is a proof-of-concept for a more general scheme that could be applied to lipids and cytokines. Our system is compatible with traditional widefield light microscopes as opposed to requiring expensive and dedicated super-resolution setups; this accessibility bodes well for establishing a significant future impact investigating multiple biological systems.

## Materials and Methods

### Reagents

Human CXCL13 and CCL19 (Almac, CAF-12 and CAF-06, respectively) singly labeled with the far-red fluorescent tag AF647 (molecular mass 1,250 Da) were stored in water at 222 µg mL^−1^. This fluorophore was chosen because of its small molecular mass, which reduces the impact of increased mass on molecular mobility, and due to its excitation at the long, lower energy wavelength range of the spectrum, which reduces sample damage. Collagen samples contained type-I collagen extracted from rat tails (51) diluted in PBS to 3.3 mg mL^−1^ and chemokine at 111 ng mL^−1^; samples were set to pH7 with the addition of NaOH. BSA labeled with 3–6 AF647 was purchased from Thermo Fisher Scientific Inc. Ficoll 400 (Sigma-Aldrich) was diluted in PBS at 0.1 g mL^−1^ to create a 10% solution of viscosity 0.005 Pa s at room temperature (52).

### Preparation of Collagen Matrix in Tunnel Slides

Samples for fluorescence microscopy were prepared in tunnel slides formed by placing two parallel lines of double-sided tape on a standard microscopy slide around 5 mm apart. A plasma-cleaned coverslip was placed on top and carefully tapped down (avoiding the imaging area) to create a water-tight tunnel.

For imaging in a collagen matrix tunnel slides were cooled to 4°C before addition of collagen and fluorescently labeled chemokines, and then incubated at 15°C for 30 min, followed by an additional 30-min incubation at 37°C. The collagen matrix was visualized using second harmonic imaging (47, 53).

Immobilized chemokine samples were prepared by incubating a plasma-cleaned coverslip in heparan sulfate (54) (50 mg mL^−1^) (Sigma-Aldrich) in PBS for 30 min. Coverslips were washed with PBS and air dried for 30 min before tunnel slide assembly then 10-nM fluorescently labeled chemokine solution in PBS was introduced to the tunnel slide and incubated in a humidity chamber for 15 min at 20°C. Excess unbound chemokine was removed with a PBS wash.

### SHIM Imaging

Second harmonic imaging microscopy was performed on a Zeiss LSM 780 MP with a Zeiss invert microscope. Excitation at 900-nm wavelength (Coherent Ultra Laser) through a plan-apochromat 63×/1.4 oil objective lens was incident on the sample. Up-converted light was collected *via* a 485-nm short pass filter onto a non-descanned detector.

### Preparation of Lymph Node Tissue Sections

Six- to eight-week-old wild-type mice (C57BL/6) were housed in BSF at the University of York. All experiments conformed to the ethical principles and guidelines approved by the University of York Institutional and Animal Care Use Committee. Popliteal Lymph Nodes were removed and excess fat or connective tissue removed with forceps. Samples were transferred to optimal cutting temperature medium (OCT, Tissue-Tek, Sakura Finetek) and snap frozen on dry ice samples and sectioned using a cryostat. 10-µm thick sections were cut and collected onto poly-l-lysine coated microscope slides. Sections were dried overnight in the dark then stored at −20°C.

Before use, lymph node sections on poly-l-lysine slides were incubated at room temperature for 30 min. Sections were hydrated in PBS for 5 min then air dried. Wax ImmEdge pen (Vector Laboratories) was used to draw a hydrophobic circle around each section to retain liquid on the section during staining. Sections were incubated in a blocking buffer of PBS 5% goat serum (Sigma) at room temperature for 1 h. To determine where B-cell follicular structures were located in the tissue we used the marker B220, a protein expressed on the surface of all murine B lymphocytes. After blocking, sections were incubated with an FITC-conjugated antibody (RA3-6B2, purchased from eBioscience) that binds specifically to B220 diluted 1:200 in blocking buffer for 1 h at room temperature. After blocking, sections were incubated with an FITC-conjugated antibody (RA3-6B2, purchased from eBioscience) that binds specifically to B220 diluted 1:200 in blocking buffer for 1 h at room temperature. Samples were washed with PBS for 3 min × 5 min.

For confocal microscopy experiments where exogeneous CXCL13-AF647/BSA-AF647 was used to measure binding to tissue, slides were stained with anti-B220 as described above followed by an incubation with 500 nM of each fluorescently labeled protein for 1 h at room temperature. Samples were then washed 1 min × 5 min in PBS. A drop of Prolong gold (Invitrogen) was added to each section, and then a No 1.5 glass coverslip (Fisher) mounted on top. The slides were incubated overnight at 4°C the next day slides were sealed using nail varnish and stored at 4°C. Immunofluorescent-stained sections were imaged using a Zeiss LSM 880 confocal microscope.

For single-molecule microscopy experiments sections were stained for B220 as described above, and then 1 µM of CXCL13-AF647 was added to the slides. Slides were incubated overnight at 4°C after which slides were washed for 30 s in PBS and a coverslip (thickness 0.1–0.17 mm, Menzel Gläser) mounted on top. Slides were then sealed and imaged.

### Stokes Model of Diffusion

For a small sphere, the diffusion coefficient is given by

$$D=\frac{k_{B}T}{6\pi \eta r}$$

where *k*_B_ is the Boltzmann constant, *T* is room temperature, η is the dynamic viscosity of the media, and *r* is the radius of a sphere, calculated assuming that the molecule is a globular protein of density 1.35 g cm^−3^ (55). The theoretical value of the diffusion coefficient of BSA in 10% Ficoll 400 was calculated using the Stokes radius of BSA of 3.48 nm (56). The Stokes radius of a dimer was assumed be double the Stokes radius of a monomer.

### Faxen’s Law

At distances probed by narrowfield fluorescence microscopy (approximately few hundred nanometers of the coverslip), the boundary effect of increased viscosity in the solution can be modeled by Faxen’s law (57):

$$\eta (h)=\eta (\infty ){\left(1+P\left(\frac{r}{h}\right)\right)}^{-1}$$

where *η*(∞) = dynamic laminar-flow viscosity in free solution, *r* is the radius of the particle, and *h* is the distance between the boundary and the center of the particle. To a fifth–order approximation:

$$P\left(\gamma \right)\approx -\frac{9\gamma }{16}+{\left(\frac{\gamma }{8}\right)}^{3}-{\left(\frac{45\gamma }{256}\right)}^{4}+{\left(\frac{\gamma }{16}\right)}^{5}$$

### SEC-MALLS of BSA-AF647

The experimental system for SEC-MALLS experiments comprised a Wyatt HELEOS-II multi-angle light scattering detector and a Wyatt rEX refractive index detector attached to a Shimadzu HPLC system (SPD-20A UV detector, LC20-AD isocratic pump system, DGU-20A3 degasser and SIL-20A autosampler). 100 µL of 2.5 mg mL^−1^ BSA-AF647 sample was run at 0.5 mL min^−1^ flow rate at room temperature through superdex S200 columns (G.E. Healthcare) for 60 min in PBS running buffer. Peaks were integrated using Astra V software and the Zimm fit method with degree 1; a value of 0.183 was used for protein refractive index increment (dn/dc).

### FCS and FRAP Microscopy

Fluorescence correlation spectroscopy and FRAP experiments were performed on a Zeiss LSM 880 microscope, using a GaAsP detector. Samples were prepared in MatTek glass bottom Petri dishes (1.5 coverglass, MatTek Corporation) and illuminated with a 633-nm wavelength laser.

For FCS the confocal volume was measured using a calibration sample of BSA-AF647 diffusion in water and constraining the diffusion coefficient to be 59 µm^2^ s^−1^ (58); this allowed the structural parameter, *s*, to be fixed at 6.6. Three repeats of 10 experiments were conducted; traces indicating the presence of multimeric clumps or proximity to the surface were excluded. Autocorrelation traces, *G*(τ), to account for transient dark states and translational diffusion were fitted with the following equation:

$$G\left(\tau \right)=1+\left(1+\frac{Te^{-\left(\frac{\tau }{\tau_{T}}\right)}}{1-T}\right)\left(\frac{1}{V_{\mathrm{eff}}<C>}\frac{1}{\left(1+\frac{\tau }{\tau_{D}}\right)}\frac{1}{\sqrt{1+{\left(\frac{1}{s}\right)}^{2}\frac{\tau }{\tau_{D}}}}\right)$$

where *T* is the triplet fraction, τ_T_ is the time constant of the dark state, *τ_D_* is the time constant of translation across the confocal volume, *V*_eff_, and < *C* > is the average concentration. The diffusion coefficient, *D*, was calculated from *τ_D_ via* the following equation:

$$D_{\mathrm{FCS}}=\frac{r_{0}{\;}^{2}}{4\tau_{D}}$$

where *r*_0_ is the spot width (0.322 µm). For FRAP microscopy a square region defined in the Zeiss Zen software was bleached with the 633-nm wavelength focused laser in an axially thin sample that was treated as being 2D. When applied to immobilized fluorophore the shape of the bleached area (see Figure 4B) was found to be well approximated as a Gaussian spot of half-width ω = 4.9 ± 0.1 µm. To measure the diffusion coefficient of BSA-AF647, 30 recovery traces [intensity (*I*) vs. time (*t*)] were acquired and fitted in the Zeiss Zen software with the single exponential equation:

$$I=I_{0}-I_{1}e^{\left(-\frac{t}{\tau_{1}}\right)}$$

where *I*_0_ is the initial intensity, *I*_1_ is drop in intensity, and *τ*_1_ is the decay constant. Thus, the diffusion coefficient, *D*, can be calculated as

$$D_{\text{FRAP}}=\frac{\omega^{2}}{{8\tau }_{1}}$$

### Single-Molecule Fluorescence Microscopy

Bespoke fluorescence microscopy was performed on an inverted microscope body (Nikon Eclipse Ti-S) with a 100× NA 1.49 Nikon oil immersion lens and illumination from a supercontinuum laser (Fianium SC-400-6, Fianium Ltd.), controlled with an acousto-optical tunable filter (AOTF) to produce excitation light centered on wavelength 619 nm (Figure S1 in Supplementary Material). A 633 nm dichroic mirror and 647 nm long-pass emission filter were used beneath the objective lens turret to exclude illumination light from the fluorescence images. The sample was illuminated with narrowfield excitation of 12 µm FWHM and an intensity of 2,300 W cm^−2^. Images were recorded on an EMCCD camera (860 iXon + , Andor Technology Ltd.) cooled to −80°C. 128 × 128 pixel images were acquired with 1.98 ms exposure times and 128 × 29 pixel image strips were acquired with 0.59 ms exposure times, both for 1,000 frames at the full EM and pre-amplifier gains of 300 and 4.6, respectively.

### Particle Tracking and Calculation of Diffusion Coefficients

All image data were recorded into .tiff files and analyzed in bespoke Matlab software. Single fluorescent molecules were identified and processed into super-resolution tracks using ADEMS code (41). The microscopic diffusion coefficient was calculated for each tracked particle from the gradient of a linear relation fitted to a plot of the mean square displacements against the four different step time intervals that can be calculated from the first four steps in a track. The microscopic diffusion coefficient distributions comprised an immobile fraction that had non-specifically adhered to the plasma-cleaned coverslip and a diffusive fraction. Microscopic diffusion coefficients were binned into histograms with bin width given by the localization precision of the immobilized (heparan sulfate) data. The probability distribution of diffusion coefficients was modeled by a gamma distribution (45, 59–61):

$$F\left(x,D,N\right)=\frac{{\left(\frac{N}{D}\right)}^{N}x^{N-1}e^{-\frac{Nx}{D}}}{\left(N-1\right)!}$$

where *N* is the number of independent steps in a track and *D* is the true diffusion coefficient. The histogram data were fitted iteratively with a two-gamma distribution to account for the mobile and immobile fractions. Initial fitting constraints conserved the number of tracks and assumed the number of independent steps in a track was 4 or less, giving a first estimation of the diffusion coefficients. Then fluorescence microscopy data with the found diffusion coefficients were simulated with and without noise, tracked, and the distribution of diffusion coefficients was fitted with the same constraints as the actual data. Fitting parameters were refined based on the results of fitting to the simulated data, and the experimental data were fitted with the refined constraints. The process of simulating the data, fitting the simulation to refine the constraints, and fitting the experimental data was repeated until the simulation represented the experimental data and the fit to the simulation data converged to the diffusion coefficient values simulated.

For immobilized spots, the *N* value was less than 1, implying that the steps are not independent. This is expected for immobile molecules as the localization precision uncertainty is larger than the diffusion distance. For mobile spots *N* was fixed at 2 as there are two steps that do not contain any common localizations when only the first four steps of a track are used.

### Simulation of Fluorescence Microscopy Data

Image datasets were simulated in bespoke MATLAB software at given diffusion coefficients using foci intensity, foci spot width, background intensity, and foci density data from real images. Foci are created at random locations in the image frame with intensity randomly chosen from a localization in an experimental dataset. The new positions of a focus in the *x-* and *y*-directions after initial generation are each determined from a Gaussian distribution centered on the previous spot location with an SD of the mean square displacement of a particle in one direction, 2*Dt*, where *D* is the simulated diffusion coefficient and *t* is the time interval between frames. To incorporate photobleaching and other effects causing truncation of trajectories foci were randomly reassigned to a new location 10% of the time; however, diffusion within a frame was not explicitly incorporated into the model beyond the use of the spot width of real localizations. The resulting image stack was used for no-noise simulations. Readout noise in the detector was incorporated in the simulations by the addition of zero mean Gaussian white noise, the intensity of which depended on the local intensity. For tissue data simulation, the mean background level and SD of the noise were taken from control data, away from autofluorescent ECM.

### Bootstrapping

Errors on the found values of the diffusion coefficients from FRAP, FCS, and single-molecule tracking were found by bootstrapping (62, 63). In this method, a randomly chosen 80% of the data is fitted in the same way as the entire data set and the SD on each parameter from ten repeats of this process is taken as the error on each fit parameter found from 100% of the data.

### Single-Molecule Imaging in Tissue Sections

Tissue sections were stained with an FITC-conjugated antibody that binds B220 (RA3-6B2, purchased from eBioscience). Tissue sections were subsequently imaged at low (1.2 μm/pixel) magnification with green illumination (wavelength 470 nm (Figure S1 in Supplementary Material), 12-µm FWHM, intensity of 875 W cm^−2^) to determine the location of the B-cell follicles, before switching to high (120 nm/pixel) magnification and red illumination to image chemokines in these areas.

### Segmentation of Tissue Sections Images

Image acquisitions in tissue contain regions of autofluorescent ECM (see Supplementary Note and Figure S5 in Supplementary Material) which are identified by the tracking software. These images must be segmented to identify tracks due to fluorescently labeled chemokine or ECM. Intensity averages of the image acquisition were top hat filtered with a structuring element of radius 4 pixels. The resulting image was converted to binary form using a threshold defined by Otsu’s method and the binary image used to enhance the ECM regions of the original image. Small holes in the thresholded region were filled by sequential erosion and dilation with a disk of radius 2 pixels as the structuring element.

### Code Availability

All our bespoke software developed is freely and openly accessible *via* https://sourceforge.net/projects/york-biophysics/.

### Statistics

Goodness-of-fit values for modeling of the distribution of microscopic diffusion coefficients were evaluated using χ^2^ tests as detailed in the text. Experimentally measured stoichiometry distributions were compared against random overlap predictions in a pairwise fashion where appropriate using Pearson’s χ^2^ test.

## Ethics Statement

All experiments conformed to the ethical principles and guidelines approved by the University of York Institutional and Animal Care Use Committee.

## Author Contributions

HM built the bespoke fluorescence microscope, overseen by ML. JC prepared biological samples overseen by MC. HM and JC performed the imaging except the confocal microscopy showing binding specificity of CXCL13-AF647 performed and analyzed by ET and ZZ. AW performed the overlap calculations. HM analyzed all other data with input from AW and ML. HM ran simulations of fluorescence data on code adapted from AW. PT oversaw FCS and FRAP microscopy. HM, AW, and ET prepared the figures with input from all authors. HM, JC, and ML wrote the manuscript with input from all authors.

## Conflict of Interest Statement

The authors declare that the research was conducted in the absence of any commercial or financial relationships that could be construed as a potential conflict of interest.

## Supplementary Material

Video S1 BSA-AF647 in PBS buffer. While some particles are tracked, many diffuse further than the tracking radius in each frame, and cannot be tracked. Video after 260 ms of imaging slowed 100×. Tracked molecules are overlaid with a white line, scale bar 1 μm.

Video S2 BSA-AF647 in 10% Ficoll 400, after 350 ms of imaging, slowed 100×. The increase in viscosity by a factor of 5.6 compared with buffer alone allows the particles to be tracked. Tracked molecules are not overlaid here to allow easier visualization of the actual level of signal and background noise, scale bar 1 μm.

Video S3 BSA-AF647 in 10% Ficoll 400, after 350 ms of imaging, slowed 100×. The same data as in video 3 is shown, with tracked molecules are overlaid with a white line, scale bar 1 μm.

Video S4 CCL19-AF647 diffusion in collagen, showing immobile population and stepwise photobleaching, slowed 100×. Tracked molecules are overlaid with a white line, scale bar 1 μm.

Video S5 CXCL13-AF647 diffusion in collagen, showing members of both the mobile and immobile populations, slowed 100×. Tracked molecules are overlaid with a white line, scale bar 1 μm.

Video S6 CXCL13-AF647 in lymph node tissue section. Tracks corresponding to both extracellular matrix and mobile chemokine are seen. 100 ms of imaging is shown, slowed 100×. Tracked molecules are overlaid with a white line, scale bar 1 μm.

Video S7 Heparan sulfate immobilized CCL19-AF647, showing a predominantly immobile population undergoing photoblinking behavior, slowed 100×. The first frames after laser illumination are included. Tracked molecules are overlaid with a white line, scale bar 1 μm.

Video S8 Heparan sulfate immobilized CXCL13-AF647, showing a predominantly immobile population, slowed 100×. Tracked molecules are overlaid with a white line, scale bar 1 μm.

## References

[1] Kienle K Lämmermann T. Neutrophil swarming: an essential process of the neutrophil tissue response. Immunol Rev (2016) 273:76–93. 10.1111/imr.12458 27558329

[2] Buckley CD Barone F Nayar S Bénézech C Caamaño J. Stromal cells in chronic inflammation and tertiary lymphoid organ formation. Annu Rev Immunol (2015) 33:715–45. 10.1146/annurev-immunol-032713-120252 25861980

[3] Drayton DL Liao S Mounzer RH Ruddle NH. Lymphoid organ development: from ontogeny to neogenesis. Nat Immunol (2006) 7:344–53. 10.1038/ni1330 16550197

[4] Pereira JP Kelly LM Cyster JG. Finding the right niche: B-cell migration in the early phases of T-dependent antibody responses. Int Immunol (2010) 22:413–9. 10.1093/intimm/dxq047 20508253 PMC2877811

[5] Dustin ML Baldari CT The immune synapse: past, present, and future. In: Baldari C Dustin M, editors. The Immune Synapse. Methods in Molecular Biology. (Vol. 1584), New York, NY: Humana Press (2017). p. 1–5. 10.1007/978-1-4939-6881-7_1 28255692

[6] Andrecka J Ortega Arroyo J Takagi Y de Wit G Fineberg A MacKinnon L Structural dynamics of myosin 5 during processive motion revealed by interferometric scattering microscopy. Elife (2015) 4:393–414. 10.7554/eLife.05413 25748137 PMC4391024

[7] Fujiwara T Ritchie K Murakoshi H Jacobson K Kusumi A. Phospholipids undergo hop diffusion in compartmentalized cell membrane. J Cell Biol (2002) 157:1071–81. 10.1083/jcb.200202050 12058021 PMC2174039

[8] Leake MC The physics of life: one molecule at a time. Philos Trans R Soc Lond B Biol Sci (2013) 368:20120248 10.1098/rstb.2012.0248 23267186 PMC3538435

[9] Miller H Zhou Z Shepherd J Wollman AJM Leake MC. Single-molecule techniques in biophysics: a review of the progress in methods and applications. Rep Prog Phys (2018) 81:24601. 10.1088/1361-6633/aa8a02 28869217

[10] Piliarik M Sandoghdar V. Direct optical sensing of single unlabelled proteins and super-resolution imaging of their binding sites. Nat Commun (2014) 5:4495. 10.1038/ncomms5495 25072241

[11] Hell SW Wichmann J. Breaking the diffraction resolution limit by stimulated emission: stimulated-emission-depletion fluorescence microscopy. Opt Lett (1994) 19:780–2. 10.1364/OL.19.000780 19844443

[12] Schneider J Zahn J Maglione M Sigrist SJ Marquard J Chojnacki J Ultrafast, temporally stochastic STED nanoscopy of millisecond dynamics. Nat Methods (2015) 12:827–30. 10.1038/nmeth.3481 26214129

[13] Balzarotti F Eilers Y Gwosch KC Gynnå AH Westphal V Stefani FD Nanometer resolution imaging and tracking of fluorescent molecules with minimal photon fluxes. Science (2017) 355:606–12. 10.1126/science.aak9913 28008086

[14] Betzig E Patterson GH Sougrat R Lindwasser OW Olenych S Bonifacino JS Imaging intracellular fluorescent proteins at nanometer resolution. Science (2006) 313:1642–5. 10.1126/science.1127344 16902090

[15] Rust MJ Bates M Zhuang X Sub-diffraction-limit imaging by stochastic optical reconstruction microscopy (STORM). Nature (2006) 3:793–5. 10.1038/NMETH929 PMC2700296 16896339

[16] Gustafsson MG. Surpassing the lateral resolution limit by a factor of two using structured illumination microscopy. J Microsc (2000) 198:82–7. 10.1046/j.1365-2818.2000.00710.x 10810003

[17] Song L Lu-Walther H-W Förster R Jost A Kielhorn M Zhou J Fast structured illumination microscopy using rolling shutter cameras. Meas Sci Technol (2016) 27:55401 10.1088/0957-0233/27/5/055401

[18] Plank M Wadhams GH Leake MC. Millisecond timescale slimfield imaging and automated quantification of single fluorescent protein molecules for use in probing complex biological processes. Integr Biol (Camb) (2009) 1:602–12. 10.1039/b907837a 20023777

[19] Reyes-Lamothe R Sherratt DJ Leake MC. Stoichiometry and architecture of active DNA replication machinery in *Escherichia coli*. Science (2010) 328:498–501. 10.1126/science.1185757 20413500 PMC2859602

[20] Juette MF Bewersdorf J. Three-dimensional tracking of single fluorescent particles with submillisecond temporal resolution. Nano Lett (2010) 10:4657–63. 10.1021/nl1028792 20939601

[21] Ashley TT Gan EL Pan J Andersson SB. Tracking single fluorescent particles in three dimensions via extremum seeking. Biomed Opt Express (2016) 7:3355–76. 10.1364/BOE.7.003355 27699104 PMC5030016

[22] Hiramoto-Yamaki N Tanaka KAK Suzuki KGN Hirosawa KM Miyahara MSH Kalay Z Ultrafast diffusion of a fluorescent cholesterol analog in compartmentalized plasma membranes. Traffic (2014) 15:583–612. 10.1111/tra.12163 24506328 PMC4265843

[23] Wieser S Moertelmaier M Fuertbauer E Stockinger H Schütz GJ. (Un)confined diffusion of CD59 in the plasma membrane determined by high-resolution single molecule microscopy. Biophys J (2007) 92:3719–28. 10.1529/biophysj.106.095398 17325009 PMC1853144

[24] van’t Hoff M de Sars V Oheim M. A programmable light engine for quantitative single molecule TIRF and HILO imaging. Opt Express (2008) 16:18495. 10.1364/OE.16.018495 18958128

[25] Axelrod D Koppel DE Schlessinger J Elson E Webb WW Mobility measurement by analysis of fluorescence photobleaching recovery kinetics. Biophys J (1976) 16:1055–69. 10.1016/S0006-3495(76)85755-4 786399 PMC1334945

[26] Axelrod D Ravdin P Koppel DE Schlessinger J Webb WW Elson EL Lateral motion of fluorescently labeled acetylcholine receptors in membranes of developing muscle fibers. Proc Natl Acad Sci U S A (1976) 73:4594–8. 10.1073/pnas.73.12.4594 1070010 PMC431558

[27] Edidin M Zagyansky Y Lardner T. Measurement of membrane protein lateral diffusion in single cells. Science (1976) 191:466–8. 10.1126/science.1246629 1246629

[28] Ehrenberg M Rigler R Rotational Brownian motion and fluorescence intensify fluctuations. Chem Phys (1974) 4:390–401. 10.1016/0301-0104(74)85005-6

[29] Magde D Elson E Webb WW Thermodynamic fluctuations in a reacting system—measurement by fluorescence correlation spectroscopy. Phys Rev Lett (1972) 29:705–8. 10.1103/PhysRevLett.29.705

[30] Adkins EM Samuvel DJ Fog JU Eriksen J Jayanthi LD Vaegter CB Membrane mobility and microdomain association of the dopamine transporter studied with fluorescence correlation spectroscopy and fluorescence recovery after photobleaching. Biochemistry (2007) 46:10484–97. 10.1021/bi700429z 17711354

[31] Calizo RC Scarlata S. Discrepancy between fluorescence correlation spectroscopy and fluorescence recovery after photobleaching diffusion measurements of G-protein-coupled receptors. Anal Biochem (2013) 440:40–8. 10.1016/j.ab.2013.04.033 23748145 PMC3770895

[32] Lagerholm BC Andrade DM Clausen MP Eggeling C. Convergence of lateral dynamic measurements in the plasma membrane of live cells from single particle tracking and STED-FCS. J Phys D Appl Phys (2017) 50:63001. 10.1088/1361-6463/aa519e 28458397 PMC5390782

[33] Macháň R Foo YH Wohland T. On the equivalence of FCS and FRAP: simultaneous lipid membrane measurements. Biophys J (2016) 111:152–61. 10.1016/j.bpj.2016.06.001 27410743 PMC4945495

[34] Guo L Har JY Sankaran J Hong Y Kannan B Wohland T. Molecular diffusion measurement in lipid bilayers over wide concentration ranges: a Comparative Study. Chemphyschem (2008) 9:721–8. 10.1002/cphc.200700611 18338419

[35] Rot A von Andrian UH. Chemokines in innate and adaptive host defense: basic chemokinese grammar for immune cells. Annu Rev Immunol (2004) 22:891–928. 10.1146/annurev.immunol.22.012703.104543 15032599

[36] Colditz IG Schneider MA Pruenster M Rot A. Chemokines at large: in-vivo mechanisms of their transport, presentation and clearance. Thromb Haemost (2007) 97:688–93. 10.1160/TH07-02-0105 17479178

[37] Schulz O Hammerschmidt SI Moschovakis GL Förster R. Chemokines and chemokine receptors in lymphoid tissue dynamics. Annu Rev Immunol (2016) 34:203–42. 10.1146/annurev-immunol-041015-055649 26907216

[38] Bennett LD Fox JM Signoret N. Mechanisms regulating chemokine receptor activity. Immunology (2011) 134:246–56. 10.1111/j.1365-2567.2011.03485.x 21977995 PMC3209565

[39] von Hundelshausen P Agten SM Eckardt V Blanchet X Schmitt MM Ippel H Chemokine interactome mapping enables tailored intervention in acute and chronic inflammation. Sci Transl Med (2017) 9. 10.1126/scitranslmed.aah6650 28381538

[40] Einstein A Über die von der molekularkinetischen Theorie der Wärme geforderte Bewegung von in ruhenden Flüssigkeiten suspendierten Teilchen. Ann Phys (1905) 322:549–60. 10.1002/andp.19053220806

[41] Miller H Zhou Z Wollman AJM Leake MC. Superresolution imaging of single DNA molecules using stochastic photoblinking of minor groove and intercalating dyes. Methods (2015) 88:81–8. 10.1016/j.ymeth.2015.01.010 25637032

[42] Robson A Burrage K Leake MC Inferring diffusion in single live cells at the single-molecule level. Philos Trans R Soc Lond B Biol Sci (2013) 368:20120029 10.1098/rstb.2012.0029 23267182 PMC3538431

[43] Llorente-Garcia I Lenn T Erhardt H Harriman OL Liu L-N Robson A Single-molecule in vivo imaging of bacterial respiratory complexes indicates delocalized oxidative phosphorylation. Biochim Biophys Acta (2014) 1837:811–24. 10.1016/j.bbabio.2014.01.020 24513194

[44] Wollman AJ Shashkova S Hedlund EG Friemann R Hohmann S Leake MC Transcription factor clusters regulate genes in eukaryotic cells. Elife (2017) 6:e27451 10.7554/eLife.27451 28841133 PMC5602325

[45] Qian H Sheetz MP Elson EL. Single particle tracking. Analysis of diffusion and flow in two-dimensional systems. Biophys J (1991) 60:910–21. 10.1016/S0006-3495(91)82125-7 1742458 PMC1260142

[46] Axelsson I Characterization of proteins and other macromolecules by agarose gel chromatography. J Chromatogr A (1978) 152:21–32. 10.1016/S0021-9673(00)85330-3

[47] Chen X Nadiarynkh O Plotnikov S Campagnola PJ. Second harmonic generation microscopy for quantitative analysis of collagen fibrillar structure. Nat Protoc (2012) 7:654–69. 10.1038/nprot.2012.009 22402635 PMC4337962

[48] Monneau YR Luo L Sankaranarayanan NV Nagarajan B Vivès RR Baleux F Solution structure of CXCL13 and heparan sulfate binding show that GAG binding site and cellular signalling rely on distinct domains. Open Biol (2017) 7:170133. 10.1098/rsob.170133 29070611 PMC5666081

[49] Elowitz MB Surette MG Wolf PE Stock JB Leibler S. Protein mobility in the cytoplasm of *Escherichia coli*. J Bacteriol (1999) 181:197–203. 9864330 10.1128/jb.181.1.197-203.1999 PMC103549

[50] Mullineaux CW Nenninger A Ray N Robinson C. Diffusion of green fluorescent protein in three cell environments in *Escherichia coli*. J Bacteriol (2006) 188:3442–8. 10.1128/JB.188.10.3442-3448.2006 16672597 PMC1482841

[51] Barnes AL Genever PG Rimmer S Coles MC Collagen–Poly(N-isopropylacrylamide) hydrogels with tunable properties. Biomacromolecules (2016) 17:723–34. 10.1021/acs.biomac.5b01251 26686360

[52] Rashid R Chee SML Raghunath M Wohland T. Macromolecular crowding gives rise to microviscosity, anomalous diffusion and accelerated actin polymerization. Phys Biol (2015) 12:34001. 10.1088/1478-3975/12/3/034001 25927668

[53] Cox G Kable E Jones A Fraser I Manconi F Gorrell MD. 3-Dimensional imaging of collagen using second harmonic generation. J Struct Biol (2003) 141:53–62. 10.1016/S1047-8477(02)00576-2 12576020

[54] Simon Davis DA Parish CR. Heparan sulfate: a ubiquitous glycosaminoglycan with multiple roles in immunity. Front Immunol (2013) 4:470. 10.3389/fimmu.2013.00470 24391644 PMC3866581

[55] Fischer H Polikarpov I Craievich AF. Average protein density is a molecular-weight-dependent function. Protein Sci (2004) 13:2825–8. 10.1110/ps.04688204 15388866 PMC2286542

[56] Ikeda S Nishinari K. Intermolecular forces in bovine serum albumin solutions exhibiting solidlike mechanical behaviors. Biomacromolecules (2000) 1:757–63. 10.1021/bm005587o 11710208

[57] Happel J Brenner H Low Reynolds Number Hydrodynamics: With Special Applications to Particulate Media. Netherlands: Springer (1981).

[58] Putnam F The Plasma Proteins: Structure, Function, and Genetic Control. 2nd ed New York, USA: Academic Press (1975).

[59] Saxton MJ. Single-particle tracking: the distribution of diffusion coefficients. Biophys J (1997) 72:1744–53. 10.1016/S0006-3495(97)78820-9 9083678 PMC1184368

[60] Vrljic M Nishimura SY Brasselet S Moerner WE McConnell HM. Translational diffusion of individual class II MHC membrane proteins in cells. Biophys J (2002) 83:2681–92. 10.1016/S0006-3495(02)75277-6 12414700 PMC1302352

[61] Zawadzki P Stracy M Ginda K Zawadzka K Lesterlin C Kapanidis AN The localization and action of topoisomerase IV in *Escherichia coli* chromosome segregation is coordinated by the SMC complex, MukBEF. Cell Rep (2015) 13:2587–96. 10.1016/j.celrep.2015.11.034 26686641 PMC5061553

[62] Asbury CL Fehr AN Block SM. Kinesin moves by an asymmetric hand-over-hand mechanism. Science (2003) 302:2130–4. 10.1126/science.1092985 14657506 PMC1523256

[63] Efron B Tibshirani R An Introduction to the Bootstrap. Chapman & Hall (1994).

