# Supplementary material for: High-Speed Single-Molecule Tracking of CXCL13 in the B-Follicle
Source: Front Immunol. 2018 May 22;9:1073. doi: 10.3389/fimmu.2018.01073 (PMC5972203; doi:10.3389/fimmu.2018.01073)
Supplement: Supplementary file 9 [file Presentation_1.PDF]

Supplementary Material

High-speed super-resolution imaging of CXCL13 in the B-Follicle

Helen Miller[1,6,+], Jason Cosgrove[2,3,4+], Adam J. M. Wollman[1,3], Emily Taylor[2,3], Zhaoukun Zhou[1,3], Peter J. O' Toole[3,5], Mark C. Coles[2,3,7,*] and Mark C. Leake[1,3,*]

[1]Department of Physics

[2]Centre of Immunology and Infection

[3]Department of Biology

[4]Department of Electronics

[5]Bioscience Technology Facility University of York, YO10 5DD, UK.

[6]Clarendon Laboratory, Department of Physics, University of Oxford, OX1 3PU, UK.

[7]Kennedy Institute of Rheumatology, University of Oxford, OX3 7FY, UK.

+ Co-first authors

* Correspondence should be addressed to M.C.L: mark.leake@york.ac.uk and M.C.C: mark.coles@kennedy.ox.ac.uk

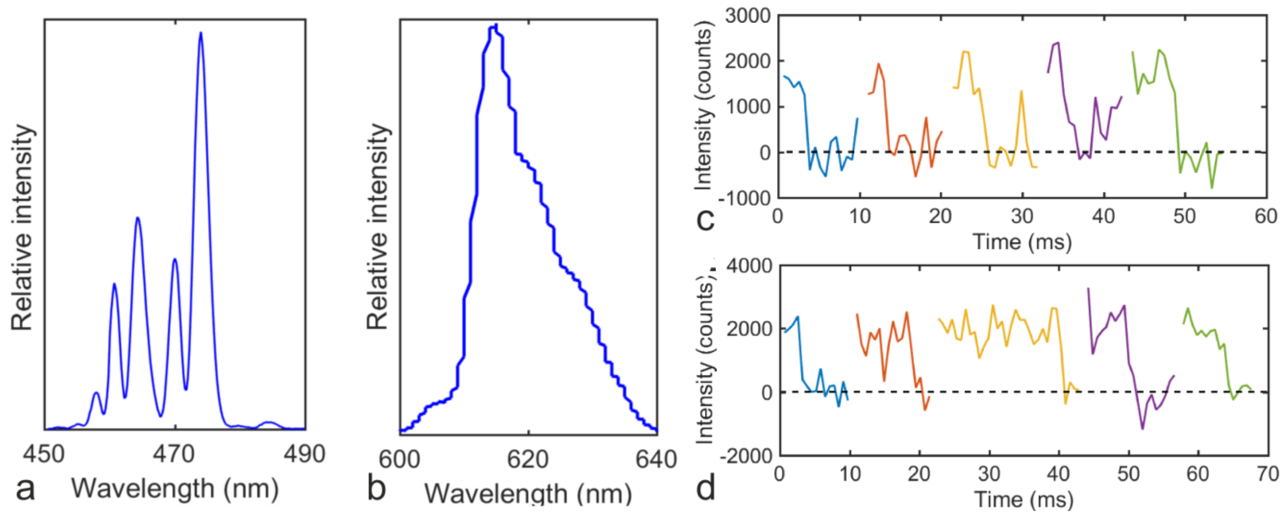

Supplementary Figure 1 Single-molecule imaging. Wavelength spectra of illumination light used for excitation of (a) FITC and (b) AF647. (c) CXCL13-AF647 and (d) CCL19-AF647 sample photobleaching steps.

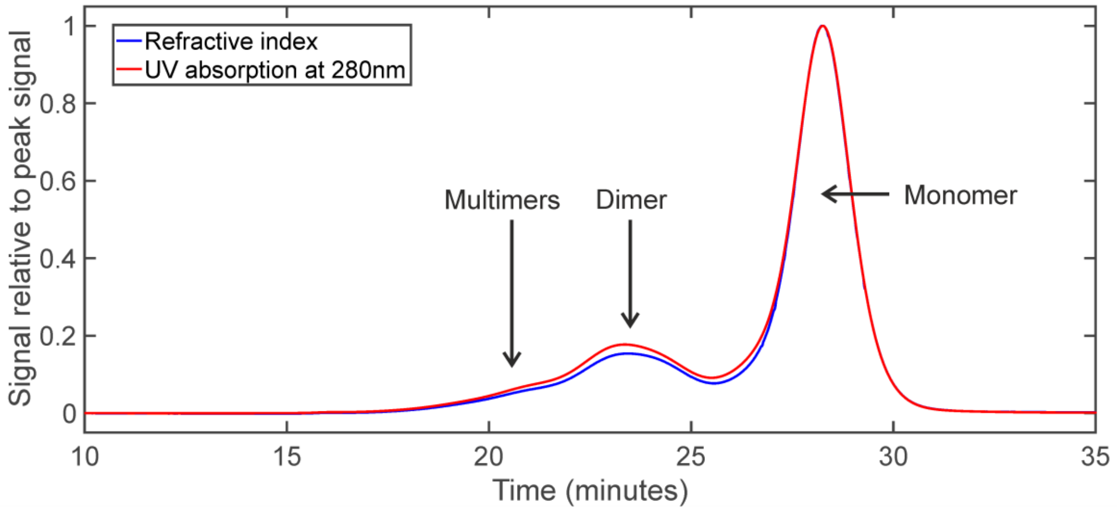

Supplementary Figure 2: SEC-MALLS on BSA-AF647. Refractive index (blue) and UV absorption at 280nm (red), scaled to the height if the monomer peak.

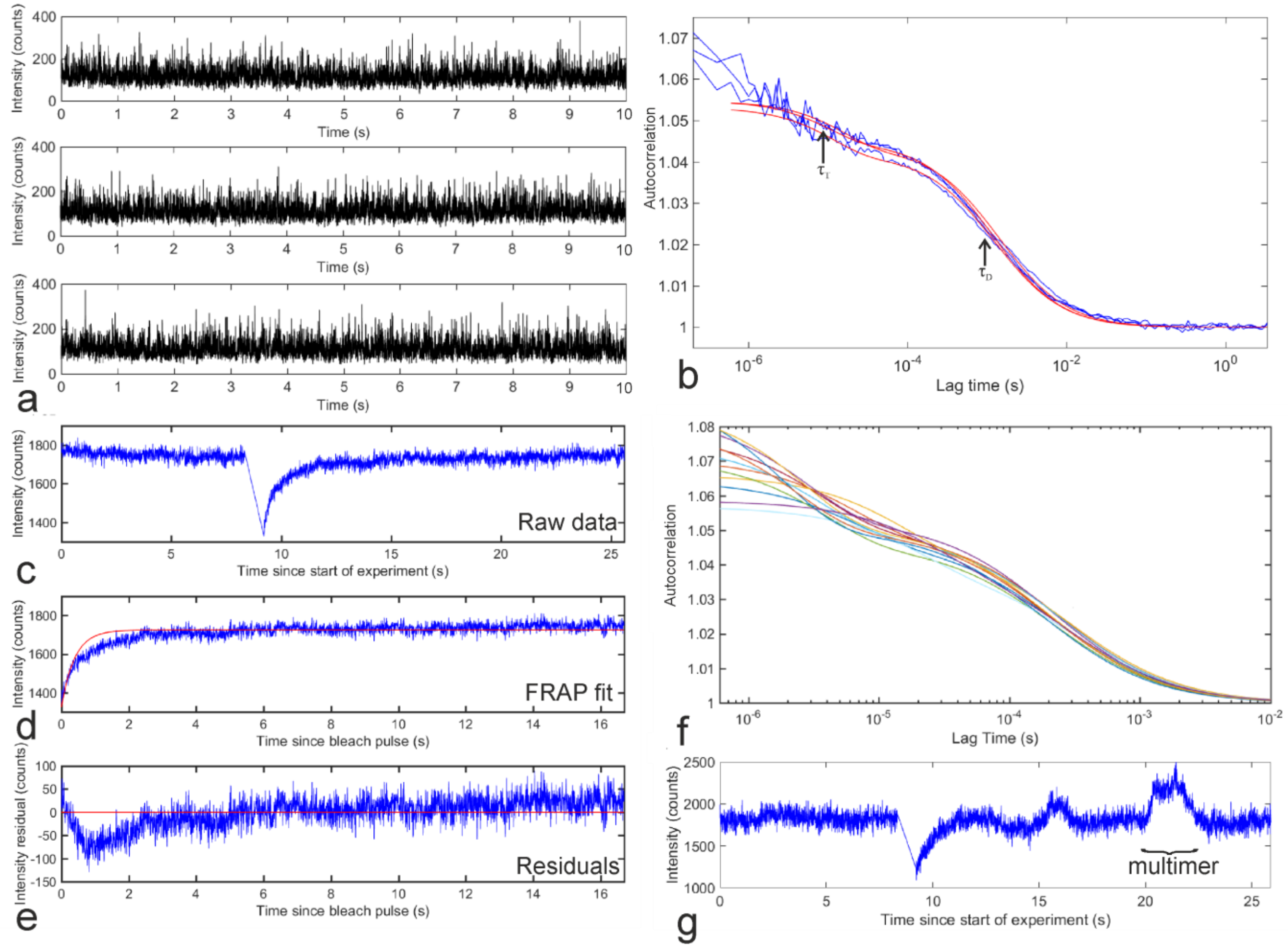

Supplementary Figure 3: FCS and FRAP. (a) Three example intensity versus time traces for FCS on BSA-AF647 in PBS buffer. (b) Example autocorrelation trace for FCS of BSA-AF647 in 10% Ficoll 400 , data shown in blue, fits shown in red. (c) Example FRAP intensity recovery trace for BSA-AF647 in 10% Ficoll 400, (d) corresponding post-bleach recovery with single exponential fit, and (e) the fitting residuals. (f) 12 example FCS autocorrelation traces for CCL19-AF647in 10% Ficoll 400 showing large variation. (g)Example FRAP trace of CXCL13 showing a large multimer diffusing into the measurement region during recovery.

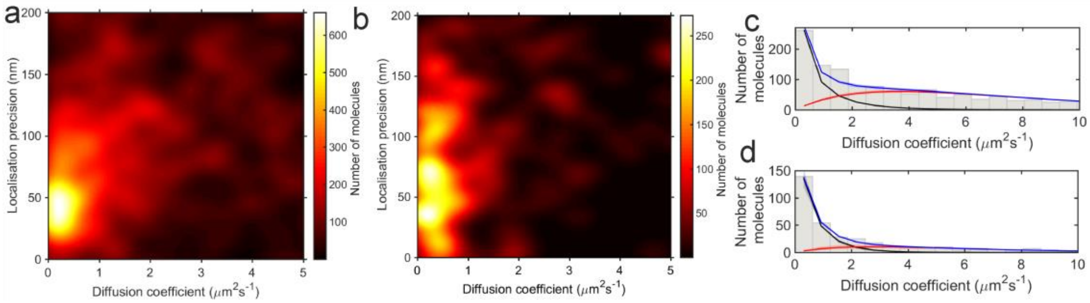

Supplementary Fig. 4: Measuring diffusion coefficients of chemokines immobilized with heparan sulfate. Heat maps of the localization precision found from the offset of the linear fit to the mean square displacement versus time plot for heparan sulfate immobilized (a) CXCL13-AF647 and (b) CCL19-AF647, the corresponding microscopic diffusion coefficient distributions are shown in (c,d); the fitted populations are shown in black for the immobile, red for the mobile and blue for the combined fit.

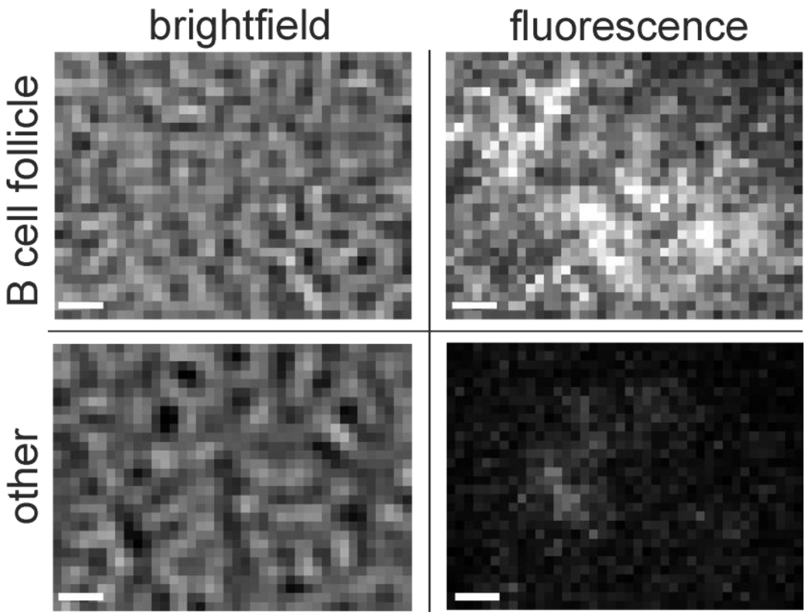

Supplementary Figure 5: Lymph node tissue section controls. Images of two regions of lymph node tissue displayed at the same contrast levels, shown in brightfield and fluorescence. Top is FITC stained B220 B cell follicle region. Bottom is unlabeled (not B cell follicle) region. Scale bars 1μm.

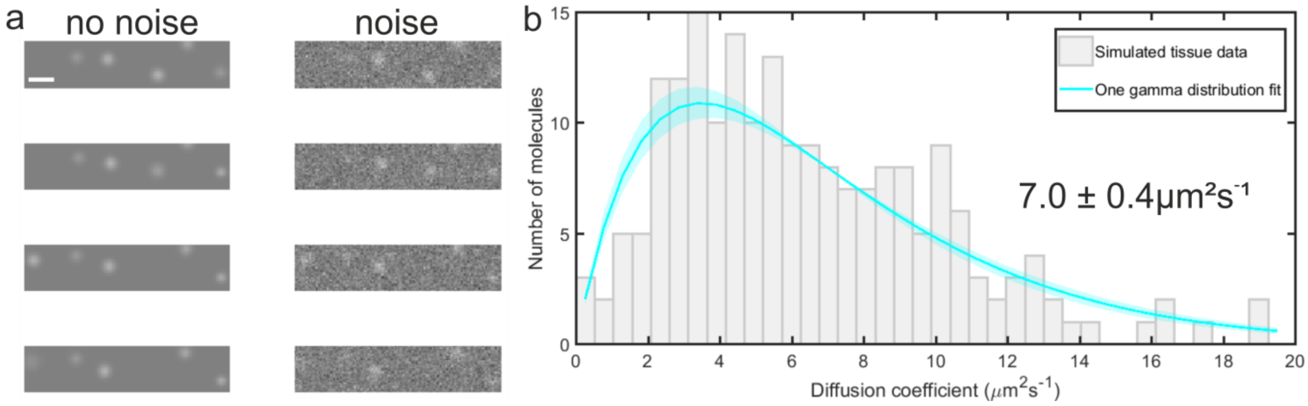

Supplementary Figure 6: Simulation of chemokine diffusion in lymph node. Images and measured diffusion coefficient distribution from a 1000 frame simulation of a diffusion coefficient of 6.6 μm^{2}s^{-1} at noise levels as measured in tissue. (a) Example images of consecutive frames from the simulation before and after the addition of noise. Scale bar 1 μm. (b) Fitted (cyan) to the diffusion coefficient distribution (gray) of simulated particles with diffusion coefficient 6.6 μm^{2}s^{-1} and the fit result of 7.0±0.4 μm^{2}s^{-1}. Shaded areas indicated one standard deviation.

Dsim ($\mu m^2 s^{-1}$)	**Fit model**	***D1*** ($\mu m^2 s^{-1}$)	**%**	***D2*** ($\mu m^2 s^{-1}$)	**%**	***D3*** ($\mu m^2 s^{-1}$)	**%**	R^2	χ^2
1.6	1 gamma fit	3.9 (3.94, 3.95)	100	-	-	-	-	0.7839	14.73
	2 gamma fit	3.1 (3.12, 3.13)	63.1 (63.09, 63.21)	11.6 (11.57, 11.61)	36.9 (36.79, 36.91)	-	-	0.9865	21.3
	3 gamma fit	0.6 (0.63, 0.64)	2.6 (2.54, 2.57)	3.2 (3.20, 3.20)	63.1 (63.04, 63.16)	11.7 (11.69, 11.72)	34.4 (34.30, 34.39)	0.9940	24.7
1.6&10	1 gamma fit	8.7 (8.63, 8.67)	100	-	-	-	-	0.2208	7.42
	2 gamma fit	3.6 (3.60, 3.61)	40.5 (40.42, 40.55)	14.7 (14.66, 14.71)	59.5 (59.45, 59.58)	-	-	0.9606	5.24
	3 gamma fit	3.6 (3.55, 3.56)	38.7 (38.65, 38.79)	13.8 (13.77, 13.82)	53.7 (53.50, 54.00)	57.5 (56.00, 59.10)	7.6 (7.40, 7.70)	0.9654	5.25

Supplementary Table 1: Results of fitting three functions to noisy simulated data. Two data sets (1.6 $\mu m^2 s^{-1}$ and a 50:50 mixture of 1.6 $\mu m^2 s^{-1}$ & 10 $\mu m^2 s^{-1}$) were fitted with 1-3 gamma distributions. The fitted values of the diffusion coefficients ($\mu m^2 s^{-1}$), the percentage of the simulated data at them, and also the R^2 and χ^2 values for each fit are given. 95% confidence bounds are indicated in brackets.

Dsim **(μm²s⁻¹)**	**Number of tracks**	***D1*** **(μm²s⁻¹)**	**% (D_1)**	***D2*** **(μm²s⁻¹)**	**N**	R^2	χ^2
1.6 & 10, no noise	1511	1.6 (1.55, 1.70)	49.2 (46.99, 51.56)	9.8 (8.99, 10.65)	2.2 (2.05, 2.29)	0.9837	4.99
1.6 & 10, Gaussian white noise	450	2.1 (1.96, 2.24)	44.9 (41.56, 48.44)	10.9 (9.77, 12.00)	3.0 (2.64, 3.33)	0.9476	3.79

Supplementary Table 2: Fitting the number of independent steps in the gamma distribution. A 50:50 mixture of particles with diffusion coefficients 1.6 $\mu m^2 s^{-1}$ and 10 $\mu m^2 s^{-1}$ was simulated with and without Gaussian white noise and fitted with a two gamma distribution, with the same fitted number of independent steps in each distribution. 95% confidence bounds are indicated in brackets.

**Peak**	**Elution time from (min)**	**Elution times until (min)**	**Mass (μg)**	**% of total mass**
**Monomer**	26.02	31.00	189.7	74.0
**Dimer**	22.02	25.98	48.5	18.9
**Higher oligomer**	18.00	21.98	16.5	6.4
**Total**	17.50	32.00	256.4	

Supplementary Table 3: Results of mass analysis on refractive index determined elution profile. Note, the total does not sum to exactly 100% since there is a small tail proportion not included in monomer or multimer peaks.

Supplementary video legends

Supplementary Video 1: BSA-AF647 in PBS buffer. Whilst some particles are tracked, many diffuse further than the tracking radius in each frame, and cannot be tracked. Video after 260ms of imaging, slowed 100x. Tracked molecules are overlaid with a white line, scale bar 1 μm.

Supplementary Video 2: BSA-AF647 in 10% Ficoll 400, after 350ms of imaging, slowed 100x. The increase in viscosity by a factor of 5.6 compared to buffer alone allows the particles to be tracked. Tracked molecules are not overlaid here to allow easier visualization of the actual level of signal and background noise, scale bar 1 μm.

Supplementary Video 3: BSA-AF647 in 10% Ficoll 400, after 350ms of imaging, slowed 100x. The same data as in video 3 is shown, with tracked molecules are overlaid with a white line, scale bar 1 μm.

Supplementary Video 4: CCL19-AF647 diffusion in collagen, showing immobile population and stepwise photobleaching, slowed 100x. Tracked molecules are overlaid with a white line, scale bar 1 μm.

Supplementary Video 5: CXCL13-AF647 diffusion in collagen, showing members of both the mobile and immobile populations, slowed 100x. Tracked molecules are overlaid with a white line, scale bar 1 μm.

Supplementary Video 6: CXCL13-AF647 in lymph node tissue section. Tracks corresponding to both extracellular matrix and mobile chemokine are seen. 100ms of imaging is shown, slowed 100x. Tracked molecules are overlaid with a white line, scale bar 1 μm.

Supplementary Video 7: Heparan sulfate immobilized CCL19-AF647, showing a predominantly immobile population undergoing photoblinking behavior, slowed 100x. The first frames after laser illumination are included. Tracked molecules are overlaid with a white line, scale bar 1 μm.

Supplementary Video 8: Heparan sulfate immobilized CXCL13-AF647, showing a predominantly immobile population, slowed 100x. Tracked molecules are overlaid with a white line, scale bar 1 μm.
